# The placental effects of trisomy for human chromosome 21 orthologs in four mouse models of Down syndrome

**DOI:** 10.1242/bio.062296

**Published:** 2025-12-18

**Authors:** Taisuke Sato, Laura L. Baxter, April D. Adams, Lauren A. Bishop, Faycal Guedj, Diana W. Bianchi

**Affiliations:** ^1^ Center for Precision Health Research, Prenatal Genomics and Therapy Section, National Human Genome Research Institute, National Institutes of Health, Bethesda, MD 20892, USA; ^2^ Department of Obstetrics and Gynecology, Division of Maternal Fetal Medicine, Baylor College of Medicine, Houston, TX 77030, USA; ^3^ Department of Molecular and Human Genetics, Baylor College of Medicine, Houston, TX 77030, USA; ^4^ Columbia University Irving Medical Center, Division of Reproductive Endocrinology and Infertility, New York, NY 10019, USA

**Keywords:** Trisomy 21, Down syndrome, Mouse models, Transcriptomics

## Abstract

Down syndrome (DS) is caused by trisomy for human chromosome 21 (*Hsa*21) and is associated with atypical neurodevelopment that begins prenatally. The developing human fetus receives nutritional support and gas exchange from the placenta, and normal placental function is essential for proper development. Placentas that sustain fetuses with trisomy 21 contain trisomic cells, but little is known about which *Hsa*21 genes are overexpressed in the placenta or their downstream molecular, cellular, and functional effects. Although access to human placentas is limited, mouse models of DS provide excellent *in vivo* systems for investigating the prenatal effects of trisomy. This study examined the placental transcriptome in four mouse models of DS: Dp(16)1/Yey, Ts65Dn, Ts66Yah, and Ts1Cje. Placental gene and protein expression analyses showed that trisomy increased the expression of *App*, *Sod1*, and *Ifnar1* in Dp(16)1/Yey, Ts65Dn, and Ts66Yah; APP and SOD1 in Dp(16)1/Yey and Ts66Yah; and IFNAR1 in Ts66Yah. Despite modest overlap of trisomy-associated gene dysregulation among these four models, altered extracellular matrix pathways in all four models and upregulation of immune system pathways in Dp(16)1/Yey and Ts66Yah were identified. Altered redox homeostasis was observed for all four models, with Ts1Cje showing distinct changes in SOD activity and antioxidant capacity in comparison to the other three models. Immunofluorescence staining revealed region-specific upregulation of APP, SOD1, and IFNAR1 in Ts66Yah trisomic placentas. This work provides a foundation for understanding the effects of trisomy for *Hsa*21 orthologs on the mouse placenta and on prenatal development.

## Teaser

**Summary:** This study identified alterations in RNA expression, protein expression, and redox homeostasis in the placentas of four mouse models of Down syndrome.

## INTRODUCTION

Down syndrome (DS), caused by trisomy for human chromosome 21 (*Hsa*21), is the most common autosomal chromosome disorder compatible with survival, occurring in 1 in 643 live births in the United States (https://www.cdc.gov/birth-defects/data-research/facts-stats/; Stallings et al., 2024). Trisomy 21 (T21) is associated with a wide variety of phenotypes that affect most organ systems including the brain, and T21 is the most frequent genetic cause of intellectual disability (Antonarakis et al., 2020). Craniofacial and brain phenotypes associated with T21 are visible prenatally, and include reduced growth, altered structure, and cellular hypotrophy (Baburamani et al., 2019; Bahado-Singh et al., 1992; Contestabile et al., 2007; Guidi et al., 2008, 2011, 2018; Guihard-Costa et al., 2006; Kitano et al., 2023; Klein and Haydar, 2022; Larsen et al., 2008; Lu et al., 2012; Patkee et al., 2020; Rotmensch et al., 1997; Stagni et al., 2018, 2019, 2020; Tarui et al., 2020; Wisniewski et al., 1984; Yun et al., 2021).

Fetal development relies on the placenta, a remarkable mammalian organ that forms and persists throughout pregnancy and provides essential functions of gas and nutrient exchange, waste removal, hormone synthesis, and immunological protection. The placenta consists of both maternal cells and cells derived from embryonic trophoblasts, thus placentas supporting fetuses with DS contain cells with T21. Evidence suggests that T21 alters placental structure and function, as previous studies have shown reduced size, hypovascular irregular villi, and increased senescence (Amiel et al., 2013; Qureshi et al., 1997; Stoll et al., 1990). Furthermore, *in vitro* analyses of cultured cytotrophoblasts from T21 placentas showed abnormal fusion into syncytiotrophoblasts, abnormal hormone production, and abnormal oxidative status (Frendo et al., 2001, 2000; Pidoux et al., 2004). The mechanisms by which T21 leads to these placental abnormalities are unknown, and the extent to which they contribute to atypical fetal development in T21 is unclear.

Mouse models are highly informative systems for analysis of many aspects of mammalian development, including placental function. Although morphological differences are present, mouse and human placentas show functional similarities that include fetal trophoblast invasion into the maternal endometrium, hemochorial placentation in which fetal trophoblasts are in direct contact with maternal blood, and syncytialization of the cellular layer contacting maternal blood (Adams et al., 2020; Woods et al., 2018). Mouse models have also been exceptional systems for molecular and functional studies of the atypical development seen in T21 (Aziz et al., 2018; Das and Reeves, 2011; Gupta et al., 2016; Herault et al., 2017; Klein and Haydar, 2022; Moore and Roper, 2007; Starbuck et al., 2014). *Hsa*21 exhibits three chromosomal regions with orthology to the mouse genome: a large segment of mouse chromosome (*Mmu*)16 and smaller regions of *Mmu*10 and *Mmu*17. This conserved synteny between the human and mouse genomes has facilitated the construction of a variety of trisomic mouse models of DS (Antonarakis et al., 2020; Herault et al., 2017; Tosh et al., 2022).

Although the placenta plays an important role in fetal development and potentially in the pathophysiology of DS, very little information exists regarding placental phenotypes in DS or mouse models of DS. We previously examined embryonic and placental weight, volume, and pathology in four genetically engineered mouse models of DS. We found that trisomy resulted in similarities and differences in morphometric measurements among these models, with the Ts66Yah model showing a notable reduction in placental size (Adams et al., 2021, 2024). The mice used for these studies – Dp(16)1/Yey, Ts65Dn, Ts66Yah, and Ts1Cje – are trisomic for overlapping *Mmu*16 segments. Dp(16)1/Yey (hereafter Dp16) carries a duplication of the full ∼23 Mb *Mmu*16 segment orthologous to *Hsa*21, from *Lipi* to *Zbtb21* (Li et al., 2007). Ts65Dn carries a small, freely segregating chromosome containing the distal *Mmu*16 segment from *Mrpl39* to *Zbtb21* fused to a proximal *Mmu*17 segment that contains 45 non-*Hsa*21-orthologous protein-coding genes (Davisson et al., 1990; Reeves et al., 1995). Ts66Yah was generated from Ts65Dn and carries the same freely segregating chromosome, but the non-*Hsa*21 orthologous genes have been removed (Duchon et al., 2022). Ts65Dn and Ts66Yah both mimic the aneuploidy present in the ∼95% of DS individuals that carry a third, freely segregating copy of *Hsa*21, but these two models show differences in prenatal gene expression and behavioral phenotypes of neonates and adults, suggesting the extraneous trisomic genetic material in Ts65Dn may contribute to its phenotypes (Guedj et al., 2023). Ts1Cje is trisomic for a smaller *Mmu*16 segment from *Sod1* to *Zbtb21* (which does not include *App*) that was translocated to distal *Mmu*12 as part of a targeted deletion of the *Sod1* locus (Sago et al., 1998). This translocation deleted seven *Mmu*12 genes, thus rendering this model monosomic for these genes that are unrelated to DS (Duchon et al., 2011).

To the best of our knowledge, no previous studies have compared the transcriptomic or biochemical differences in the placentas of multiple mouse models of DS. In this study, we examined placental gene expression changes that resulted from trisomy at embryonic day (E)18.5 in Dp16, Ts65Dn, Ts66Yah, and Ts1Cje mice. Enriched pathway analyses showed that all four models exhibited altered extracellular network pathways, and Dp16 and Ts66Yah showed notable enrichment of immune system pathways. Given the upregulation of the trisomic genes superoxide dismutase 1 (*Sod1*), amyloid beta precursor protein (*App*), and interferon (alpha and beta) receptor 1 or 2 (*Ifnar1* or *Ifnar2*, respectively) in Dp16, Ts65Dn, and Ts66Yah, and the potential roles of these genes in human DS phenotypes (Barone et al., 2018; Galbraith et al., 2023; Lott et al., 2006; Malle and Bogunovic, 2021), we examined redox homeostasis, amyloid metabolism, and inflammatory factor status across all four mouse models. Additionally, trisomy-associated differences in SOD1, APP, and IFNAR1/2 expression were quantitated in Ts66Yah placentas by immunofluorescence, revealing region-specific upregulation. This examination of the effects of trisomy for mouse orthologs of *Hsa*21 on gene and protein expression in the placenta across multiple mouse models of DS provides a foundation for future studies regarding the relevance of this important organ to developmental phenotypes.

## RESULTS

### Dysregulated placental gene expression in four mouse models of Down syndrome

**Fig. 1.**
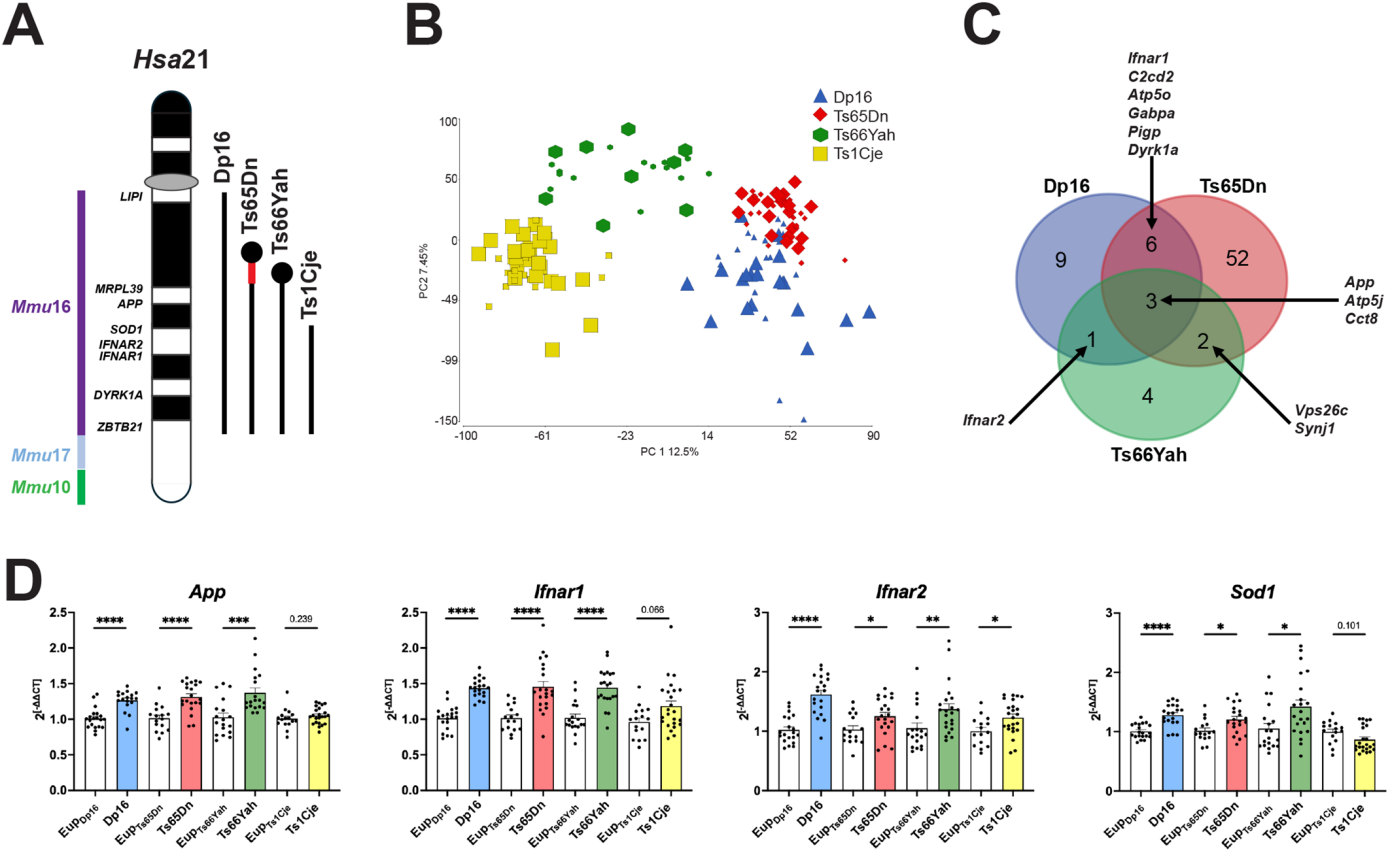
**Placental transcriptomic analyses of four mouse models of Down syndrome.** (A) Chromosomal regions of conserved synteny with human chromosome 21 (*Hsa*21) are present in the mouse genome on mouse chromosome (*Mmu*)16, 17, and 10 (left), and the trisomic regions in Dp16, Ts65Dn, Ts66Yah, and Ts1Cje models are shown (right). Dp16 is a segmental duplication model and is trisomic for the full *Mmu*16 region orthologous to *Hsa*21 (141 protein-coding genes from *Lipi* to *Zbtb21*, including a 37 gene keratin cluster). Ts65Dn and Ts66Yah carry an extra chromosome and thus are aneuploid, illustrated by circles representing centromeres. Both Ts65Dn and Ts66Yah are trisomic for 129 protein-coding genes (from *Mrpl39* to *Zbtb21*, including keratins), but Ts65Dn is trisomic for an additional *Mmu*17 region that is non-orthologous to *Hsa*21 and encodes 45 protein-coding genes (red segment). Ts1Cje is a translocation model and is trisomic for 71 protein-coding genes (*Sod1* to *Zbtb21*). Ts1Cje also contains a deletion of seven distal *Mmu*12 genes. Note that Ts1Cje is disomic for *App* and functionally disomic for *Sod1*, as the third *Sod1* locus is disrupted and does not encode functional protein*.* Protein-coding gene numbers for trisomic *Mmu*16 regions from GENCODE release M37. (B) Principal component analysis plot of all microarray data. Ts1Cje (yellow) and Ts66Yah (green) clustered separately, and Ts65Dn (red) and Dp16 (blue) clusters showed a small amount of overlap. None of the models showed subclusters based on genotype. Small symbols=euploid, large symbols=trisomic. Each symbol represents an individual placenta. (C) Venn diagram of overlapping DEGs in Dp16, Ts65Dn, and Ts66Yah. Venn diagram generated using http://bioinformatics.psb.ugent.be/webtools/Venn/. (D) RT-qPCR analyses of trisomic genes *Sod1*, *App*, *Ifnar1* and *Ifnar2*. Values shown are normalized with *Gapdh*; Fig. S1 shows values from duplicate experiments normalized to *Hprt.* Sample numbers were as follows: Dp16=23 trisomic, 22 euploid; Ts65Dn=23 trisomic, 18 euploid; Ts66Yah=22 trisomic, 21 euploid; Ts1Cje=26 trisomic, 18 euploid. All samples were run in triplicate, and the average for each sample is plotted and was used for statistical analyses. Error bars=s.e.m. Unpaired, two-tailed *t*-tests were performed for datasets with normal distribution, and two-tailed Mann–Whitney tests for datasets with non-normal distribution. *P<0.05, **P<0.01, ***P<0.001, ****P<0.0001.

To examine the effects of trisomy for mouse orthologs of *Hsa*21 on the placenta, high-throughput microarray analyses were performed on E18.5 euploid and trisomic placentas from four mouse models of DS: Dp16, Ts65Dn, Ts66Yah, and Ts1Cje (Fig. 1A). Principal component analysis (PCA) using expression data from all microarray probes (Fig. 1B) showed that Ts66Yah and Ts1Cje samples clustered independently, while Ts65Dn and Dp16 showed partially overlapping clusters. None of the four models showed separation by PCA according to genotype, indicating moderate transcriptomic differences between euploid and trisomic placentas.

Concordant with this finding, few differentially expressed genes [DEGs; false discovery rate (FDR) adjusted *P*-value <0.1 and fold change >|1.2|] were identified between euploid and trisomic placentas for each model: 19 genes for Dp16, 63 genes for Ts65Dn, ten genes for Ts66Yah, and four genes for Ts1Cje (Table 1; Table S1). Ts1Cje showed no DEG overlap with the other three models. The shared DEGs among Dp16, Ts65Dn, and Ts66Yah were all trisomic *Mmu*16 genes, and included *App*, chaperonin containing TCP1 subunit 8 (*Cct8*), and ATP synthase peripheral stalk subunit F6 (*Atp5pf*, also known as *Atp5j*; Fig. 1C). In addition, Dp16, Ts65Dn, and Ts66Yah showed differential expression of *Ifnar1* and/or *Ifnar2* (Fig. 1C), which encode proteins that heterodimerize to form the IFNAR receptor.

**Table 1.** Differentially expressed genes and marginally dysregulated genes in placentas of four mouse models of Down syndrome

Model	Differentially expressed genes	MDGs	Upregulated autosomal MDGs	Downregulated autosomal MDGs	Sex chromosome and unclassified MDGs
Dp16	19	928	426 (45.9%)	443 (47.7%)	59 (6.4%)
Ts65Dn	63	965	501 (51.9%)	418 (43.3%)	46 (4.8%)
Ts66Yah	10	835	535 (64.1%)	260 (31.1%)	40 (4.8%)
Ts1Cje	4*	596	235 (39.4%)	332 (55.4%)	30 (5.0%)

*Sod1 was not included due to expression of aberrant *Sod1* transcript in Ts1Cje. MDG, marginally dysregulated gene.

Comparison of the DEG lists with a previous single-nuclei RNA-sequencing (RNA-Seq) study of the developing euploid mouse placenta (Marsh and Blelloch, 2020) found that several DEGs showed enriched expression in specific cell types of the labyrinth, a rodent-specific placental structure in which transport occurs between fetal and maternal blood. *App* expression identified sinusoidal trophoblast giant cell (sTGC) precursors and mature sTGCs of the labyrinth, indicating maintenance of *App* expression throughout placental development in this cell type. *Ifnar2* was a marker of syncytiotrophoblast-I (SynT-I) precursors, and *Cct8* was a marker of syncytiotrophoblast-II (SynT-II) precursors, but these genes did not mark mature SynT-1 cells or SynT-II cells, respectively. Therefore, *Ifnar2* and *Cct8* expression normally decreases as typical placental development progresses, but this decrease may not occur in trisomy.

Expression of *App*, *Ifnar1* and *Ifnar2* was validated by reverse transcription (RT)-quantitative PCR (qPCR), and these results paralleled those of microarray analyses (Fig. 1D; Fig. S1). *App* showed upregulation as a result of trisomy in Dp16, Ts65Dn, and Ts66Yah, while Ts1Cje showed no change in expression, as expected because this model is disomic for *App* (Fig. 1A). Even though the *Ifnar1* locus is triplicated in all four models, only Dp16, Ts65Dn, and Ts66Yah showed *Ifnar1* upregulation in trisomic placentas. *Ifnar2* showed upregulation in Dp16 and Ts66Yah trisomic placentas, and a trend towards upregulation in Ts65Dn and Ts1Cje trisomic placentas (Fig. 1D; Fig. S1).

*Sod1* showed upregulation in Ts1Cje placentas by microarray analyses, even though these mice carry two normal copies of *Sod1.* A third, disrupted *Sod1* locus (in which a neomycin resistance cassette deleted exons 3 and 4 but left exons 1, 2, and 5 intact) is present near the Ts1Cje translocation breakpoint, and this locus generates an aberrant transcript that does not encode functional SOD1 (Huang et al., 1997; Sago et al., 1998). Since microarray analyses may not have distinguished between the abnormal and full-length *Sod1* mRNA transcripts, qPCR was performed using an assay spanning exon 4 of *Sod1* to selectively amplify full-length transcript*.* The results showed a trend towards lower expression of full-length *Sod1* in trisomic Ts1Cje placentas (Fig. 1D; Fig. S1). This confirmed that the microarray results showing upregulation of *Sod1* were measuring both aberrant and full-length transcripts; thus, *Sod1* was excluded from the Ts1Cje DEG list. In contrast, Dp16, Ts65Dn, and Ts66Yah, which each harbor three full copies of the *Sod1* locus, showed upregulation of *Sod1* in trisomic placentas by qPCR (Fig. 1D; Fig. S1).

**Fig. 2.**
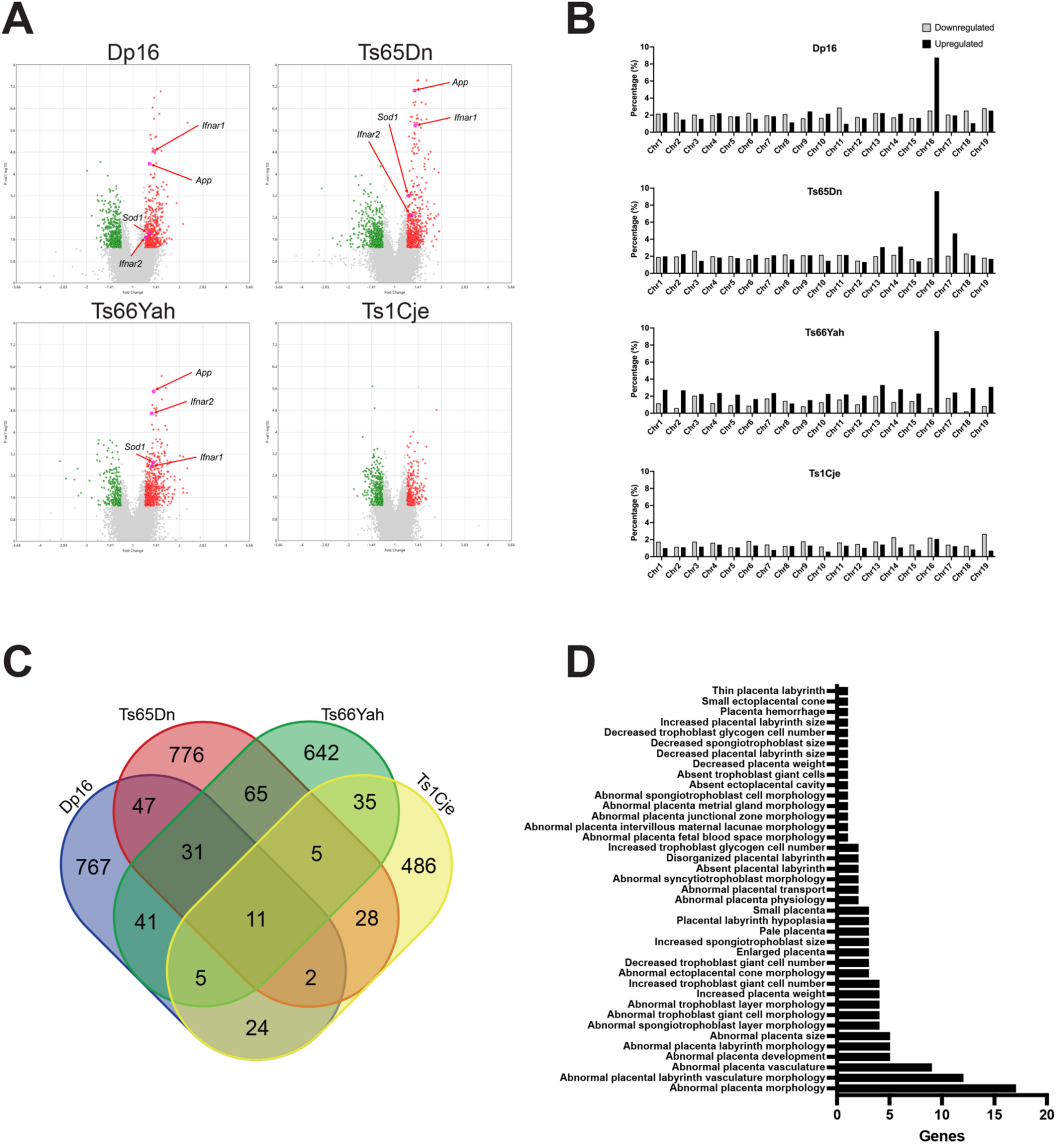
**Comparison of marginally dysregulated placental genes in four mouse models of Down syndrome.** (A) Volcano plots highlighting upregulated (red) and downregulated (green) marginally dysregulated genes (MDGs) in trisomic placentas of each model. The *Mmu*12-encoded gene *Dnah11* was excluded from the Ts1Cje plot because it showed high expression due to the translocation of distal *Mmu*16 to *Mmu*12. (B) MDGs show genome-wide distribution in each model. *y*-axes show the percentage of microarray probes that are MDGs on each chromosome. Higher percentages of MDGs on *Mmu16* in all models and on *Mmu17* in Ts65Dn correspond with trisomic genomic regions. (C) Venn diagram illustrating minimal MDG overlap in all four models. (D) Multiple mouse placental phenotypes are associated with MDGs. Queries of Mouse Genome Informatics (https://www.informatics.jax.org/batch) using the four MDG lists from each model (2947 total genes) identified 54 genes associated with 39 mammalian phenotype IDs (MPs) related to the placenta or trophoblasts. Individual gene associations with each MP are shown in the bar graph. Some genes were associated with multiple MPs (121 gene-MP associations in total; Table S3C).

Application of a less stringent cutoff of unadjusted *P*-value <0.05 and fold change >|1.2| generated more extensive gene lists that we named marginally dysregulated genes (MDGs), and these MDG sets were used for all subsequent analyses (Table 1; Table S1). Upregulated MDGs showed more significant *P-*values than downregulated MDGs in Dp16, Ts65Dn and Ts66Yah (Fig. 2A). Higher percentages of MDGs were present on *Mmu*16 for all four models and on *Mmu*17 for Ts65Dn, and most MDGs were upregulated, concordant with their trisomic genomic regions (Fig. 2B; Table S2). All four MDG lists showed genome-wide distribution (Fig. 2B), indicating that partial trisomy for *Mmu*16 (and *Mmu*17 in Ts65Dn) gave rise to transcriptomic changes throughout the genome. Intriguingly, Ts1Cje showed less differential expression of trisomic *Mmu*16 genes in comparison to the other three models, as only 13% of trisomic *Mmu*16 genes were upregulated MDGs (nine MDGs/71 trisomic *Mmu*16 genes). In contrast, 32-43% of the trisomic *Mmu*16 genes in the other three models were MDGs (Dp16=32%, 45 MDGs/141 trisomic genes; Ts65Dn=38%, 49 MDGs/129 trisomic genes; Ts66Yah=43%, 55 MDGs/129 trisomic genes). The smaller percentage of differentially expressed trisomic genes in Ts1Cje suggests that the transcriptomic effects of trisomy on the placenta may be reduced in this model.

Eleven MDGs were shared among the four models: eight trisomic *Mmu16* genes – *C2cd2*, *Cct8*, *Hlcs*, *Hmgn1*, *Itsn1*, *Ltn1*, *Usp16*, *Zbtb21* – and three non-trisomic genes, the C-type lectin receptor *Clec1b* and two prolactin genes, *Prl4a1* and *Prl8a1* (Fig. 2C, Table 2). *Prl4a1* and *Prl8a1* are members of the mouse prolactin/hormone gene family and show placenta-specific expression. *Prl4a1* is essential for an adaptive response to hypoxia in pregnancy and is normally downregulated by E18.5 (Simmons et al., 2008), while *Clec1b* is involved in platelet activation that regulates neurovascular development as well as inflammation/immune responses (Finney et al., 2012; Lowe et al., 2015; Navarro-Núñez et al., 2013). Significant upregulation of *Prl4a1* and *Clec1b* was confirmed by qPCR in Dp16, Ts65Dn, and Ts66Yah trisomic placentas, and a trend towards upregulation was seen in Ts1Cje (Fig. S1). Forty-two shared MDGs were present among Dp16, Ts65Dn, and Ts66Yah: 29 trisomic *Mmu*16 genes and 13 genes from non-trisomic regions (Fig. 2C, Table 2). Several of these shared MDGs were identified in previous gene expression studies in Ts1Cje and human placentas: *C2cd2*, *Hlcs*, *Hmgn1*, and *Itsn1* were upregulated in the Ts1Cje placenta (Pennings et al., 2011), and *APP*, *ETS2*, *ITSN1*, *HMGN1*, and *USP16* were upregulated in human placentas (Bianco et al., 2016; Rozovski et al., 2007; Wong et al., 2018).

**Table 2.** Shared differentially expressed genes and marginally dysregulated genes among placentas of four mouse models of Down syndrome

Mouse models	Chromosome	Marginally dysregulated genes*
Dp16, Ts65Dn, Ts66Yah, Ts1Cje	Trisomic *Mmu*16 region	*C2cd2*, *Cct8*, *Hlcs*, *Hmgn1*, *Itsn1*, *Ltn1*, *Usp16*, *Zbtb21*
	Non-trisomic regions	*Clec1b*, *Prl4a1*, *Prl8a1*
Dp16, Ts65Dn, Ts66Yah	Trisomic *Mmu*16 region	***App***, ***Atp5j***, ***Atp5o***, *B3galt5*, *Bace2*, ***C2cd2***, ***Cct8***, *Donson*, ***Dyrk1a***, *Ets2*, ***Gabpa***, *Hlcs*, *Hmgn1*, ***Ifnar1***, ***Ifnar2***, *Itsn1*, *Mis18a*, *Mrpl39*, *Mx2*, ***Pigp***, *Ltn1*, *Rcan1*, *Ripk4*, *Sod1*, ***Synj1***, *Ttc3*, *Usp16*, ***Vps26c***, *Wrb*, *Zbtb21*
	Non-trisomic regions	*Angpt2*, *Arhgap9*, *Bcl11a*, *Chrdl2*, *Clec1b*, *Clic3*, *Pcsk6*, *Prl4a1*, *Prl8a1*, *Rtp4*, *Smpx*, *Vcan*

*Shared differentially expressed genes among Ts65Dn, Ts66Yah, and/or Dp16 are in bold.

### Embryonic lethal and placental phenotypes of dysregulated genes

Comprehensive mouse screens have shown that embryonic lethal mutations frequently give rise to placental abnormalities (Mohun et al., 2013), and placenta-specific gene knockouts have been shown to be partially or fully causative for embryonic heart defects (Adams et al., 2000; Barak et al., 1999; Maruyama et al., 2016; Radford et al., 2023; Torregrosa-Carrion et al., 2021). This suggests that placental abnormalities may be a previously unappreciated source of developmental defects. A query of Mouse Genome Informatics (MGI) for mammalian phenotype annotation (https://www.informatics.jax.org/batch) using a master MDG list (generated by combining all four MDG lists) showed that 9.5% of these genes (279/2947) were associated with prenatal lethality (Table S3A). These included the shared DEGs *Atp5o*, *Cct8*, *Dyrk1a*, *Gabpa*, and *Vps26c*. This MGI query also identified 54 MDGs associated with placental phenotypes, the majority of which were related to placental morphology and structure (Fig. 2D; Table S3B,C). This gene list included the shared MDGs *Prl4a1*, *Ets2*, and *Vcan* (encoding the extracellular matrix protein versican). Intriguingly, 21 (39%) of these placental phenotype-associated MDGs were also associated with abnormal heart or brain phenotypes, including *Baiap2*, *Dlc1*, *Erg*, *Gpc3*, *Mecom*, *Myh10*, and *Vcan* (Table S3D).

### Enriched pathway analyses

Metascape enriched pathway analyses performed for the MDG sets from Dp16, Ts65Dn, Ts66Yah, and Ts1Cje revealed both similarities and differences (Fig. S2, Table S4). The most significant functional pathway clusters for Dp16 related to circulatory system function, detection of stimulus, and extracellular matrix/cytokine signaling; for Ts65Dn to extracellular matrix, sensory perception, and metabolic processes; for Ts66Yah to immune system/inflammatory response and extracellular matrix; and for Ts1Cje to circulatory system function and G protein-coupled receptor signaling. Both *App* and *Sod1* were present in multiple pathway clusters for Dp16, Ts65Dn, and Ts66Yah (*App*=4/20, 9/20, 8/20 and *Sod1=*6/20, 3/20, 7/20 in Dp16, Ts65Dn, and Ts66Yah, respectively, Table S4). *Ifnar1* and *Ifnar2* dysregulation was present in four pathway clusters in both Dp16 and Ts66Yah, but in only one Ts65Dn cluster.

**Fig. 3.**
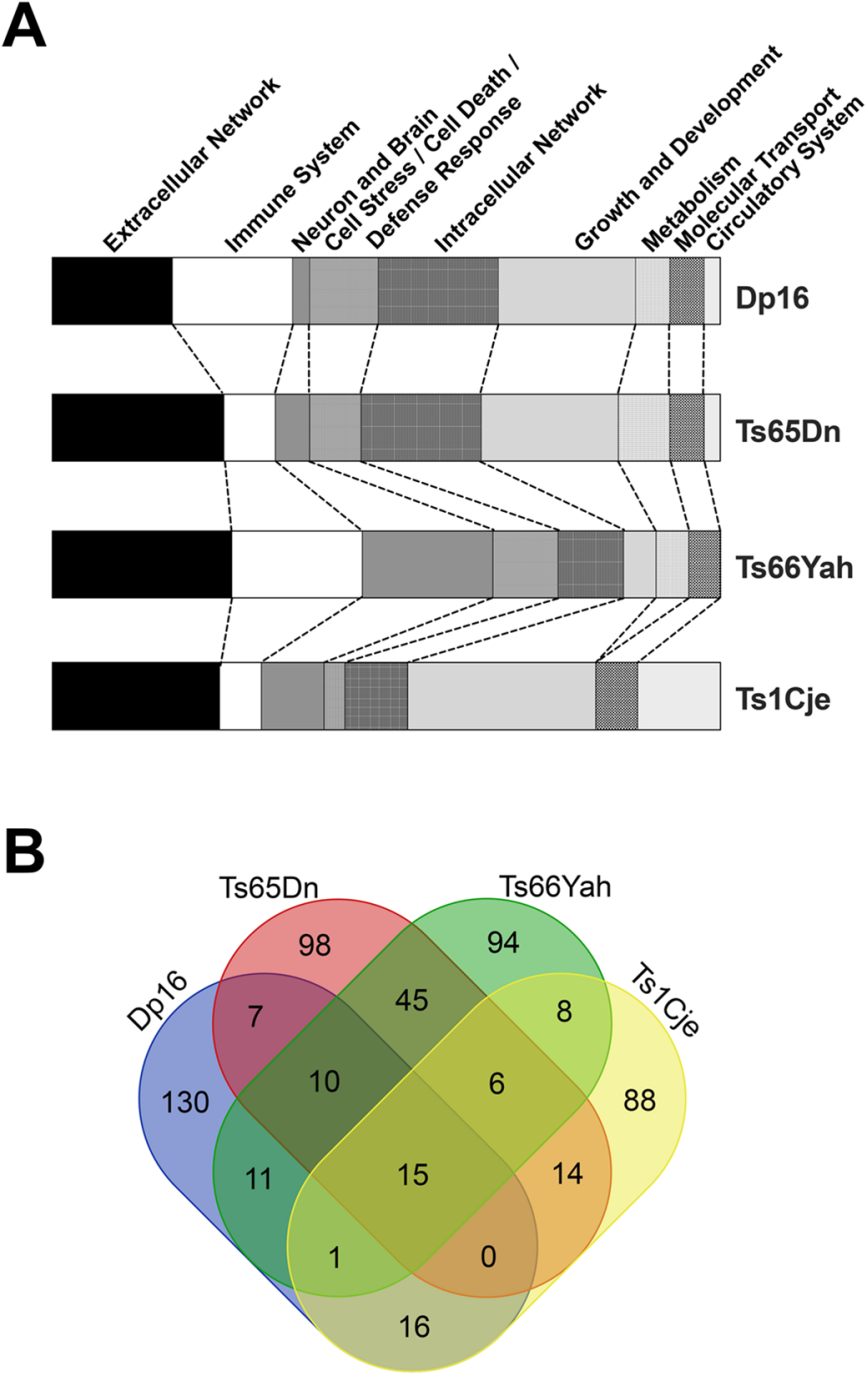
**Metascape enrichment analyses of marginally dysregulated genes in placentas of four mouse models of Down syndrome.** (A) Heatmaps illustrating higher order aggregation of the enriched pathways for each model. All pathways within the top 20 clusters for each model were manually re-categorized into nine biological groups (shown at top). (B) Venn diagram showing overlap of enriched pathways in the 20 most significant functional clusters for each model. Fifteen pathways are shared among the four models, and ten additional pathways are shared among Ts65Dn, Dp16, and Ts66Yah.

To compare enriched pathways across the four models, Metascape pathways within the top 20 clusters for each model were manually re-categorized into nine functional groups (Fig. 3A; Table S4). This broad functional aggregation showed that all four models had high percentages of extracellular network pathways. In addition, Ts66Yah and Dp16 had higher percentages of immune system pathways than Ts65Dn and Ts1Cje. Ts66Yah showed few pathways related to growth and development, while the other three models showed high percentages of these pathways.

Shared enriched pathways among the models reflected these broad functional categories. The 15 pathways that were shared among the four mouse models (Fig. 3B, Table 3) grouped into three functional clusters that were related to the extracellular matrix/matrisome, detection of extracellular/sensory stimulus, and the positive regulation of immune response/response to stimulus. Examination of shared pathways among Dp16, Ts65Dn, and Ts66Yah expanded this to six functional clusters (Table 3). The additional three clusters were related to the inflammatory response, regulation of immune effector process, and fatty acid/arachidonic acid metabolism. These results suggest that placental trisomy alters extracellular signaling pathways in all four models and immune system and metabolic pathways in Dp16, Ts65Dn, and Ts66Yah.

**Table 3.** Shared enriched IPA pathways in placentas of four mouse models of Down syndrome, grouped according to pathway clustering for each model

Metascape functional cluster	Ontology term	Pathway/term description	Dp16 −Log10(*p*)	Ts65Dn −Log10(*p*)	Ts66Yah −Log10(p)	Ts1Cje −Log10(p)
1	M5885	Naba matrisome associated	10.19	11.65	7.79	5.63
	M5883	Naba secreted factors	5.37	5.87	3.60	3.28
2	GO:0007606	Sensory perception of chemical stimulus	10.15	8.56	5.47	2.91
	GO:0007608	Sensory perception of smell	7.29	5.83	4.03	2.85
	R-HSA-9709957	Sensory perception	7.90	9.85	3.62	2.27
	R-HSA-381753	Olfactory signaling pathway	6.89	6.37	3.37	2.34
	GO:0051606	Detection of stimulus	10.56	9.06	3.33	5.27
	GO:0009593	Detection of chemical stimulus	8.07	7.08	3.31	2.36
	hsa04740	Olfactory transduction	6.40	6.38	3.28	2.89
	R-HSA-9752946	Expression and translocation of olfactory receptors	6.57	6.05	3.07	2.42
	GO:0050906	Detection of stimulus involved in sensory perception	8.21	8.70	2.89	2.76
	GO:0050911	Detection of chemical stimulus involved in sensory perception of smell	6.38	5.43	2.80	2.32
	GO:0050907	Detection of chemical stimulus involved in sensory perception	7.29	6.79	2.96	
3	GO:0032103	Positive regulation of response to external stimulus	6.79	5.90	6.19	2.22
	GO:0050778	Positive regulation of immune response	3.11	3.10	4.37	3.96
	GO:0002833	Positive regulation of response to biotic stimulus	3.57	2.38	4.04	2.23
	GO:0031349	Positive regulation of defense response	5.34	3.66	3.50	
	GO:0006954	Inflammatory response*		5.37		
4	GO:0006954	Inflammatory response*	8.05		7.50	
5	GO:0002697	Regulation of immune effector process	2.75	2.08	6.55	
6	GO:0033559	Unsaturated fatty acid metabolic process	3.42	2.80	3.99	
	GO:0120254	Olefinic compound metabolic process	3.38	3.37	3.41	
	R-HSA-2142753	Arachidonic acid metabolism	3.09	4.69	2.73	
	GO:0001676	Long-chain fatty acid metabolic process	2.66	3.88	2.54	
	GO:0006690	Icosanoid metabolic process	3.04	2.48	2.37	
	GO:0019369	Arachidonic acid metabolic process	2.41	3.13	2.05	

*Inflammatory response clustered separately in Dp16 and Ts66Yah but clustered together with other pathways in Ts65Dn.

Ingenuity pathway analysis (IPA) using the four MDG lists (Table S5) showed many similarities to Metascape analyses. Dp16, Ts65Dn, and Ts66Yah all showed enrichment of multiple pathways related to cellular immune response and immune system, and many of these pathways were predicted to be upregulated in trisomic placentas (Fig. S3). These three models showed enrichment of ‘Interferon Signaling’, ‘Role of JAK1, JAK2 and TYK2 in Interferon Signaling’, ‘Neuroinflammation Signaling Pathway’, and ‘Pathogen Induced Cytokine Storm Signaling Pathway’, with Dp16 and Ts66Yah showing more significant *P*-values and greater upregulation than Ts65Dn. Of note, most of the significantly dysregulated canonical pathways for Ts1Cje showed predicted downregulation in comparison to euploid (Fig. S3), while the other three models showed either similar levels of up- and downregulation (Dp16) or more upregulation (Ts65Dn, Ts66Yah).

IPA-predicted upstream regulators for all four models included activation by the proinflammatory cytokine tumor necrosis factor (TNF; Table S6), of interest because individuals with DS show evidence of increased TNFα signaling in blood (Sullivan et al., 2017; Zhang et al., 2017). Dp16 and Ts66Yah shared 30 upstream regulators, many of which showed predicted activation of inflammatory pathways (Table S6). These included upregulation of lipopolysaccharide, IFNAR, IFN alpha, IFN beta, and STAT1 pathways, as well as upregulation of innate immune signaling via STING1 upregulation and TREX1 downregulation.

### Expression of SOD1, APP, and IFNAR1

**Fig. 4.**
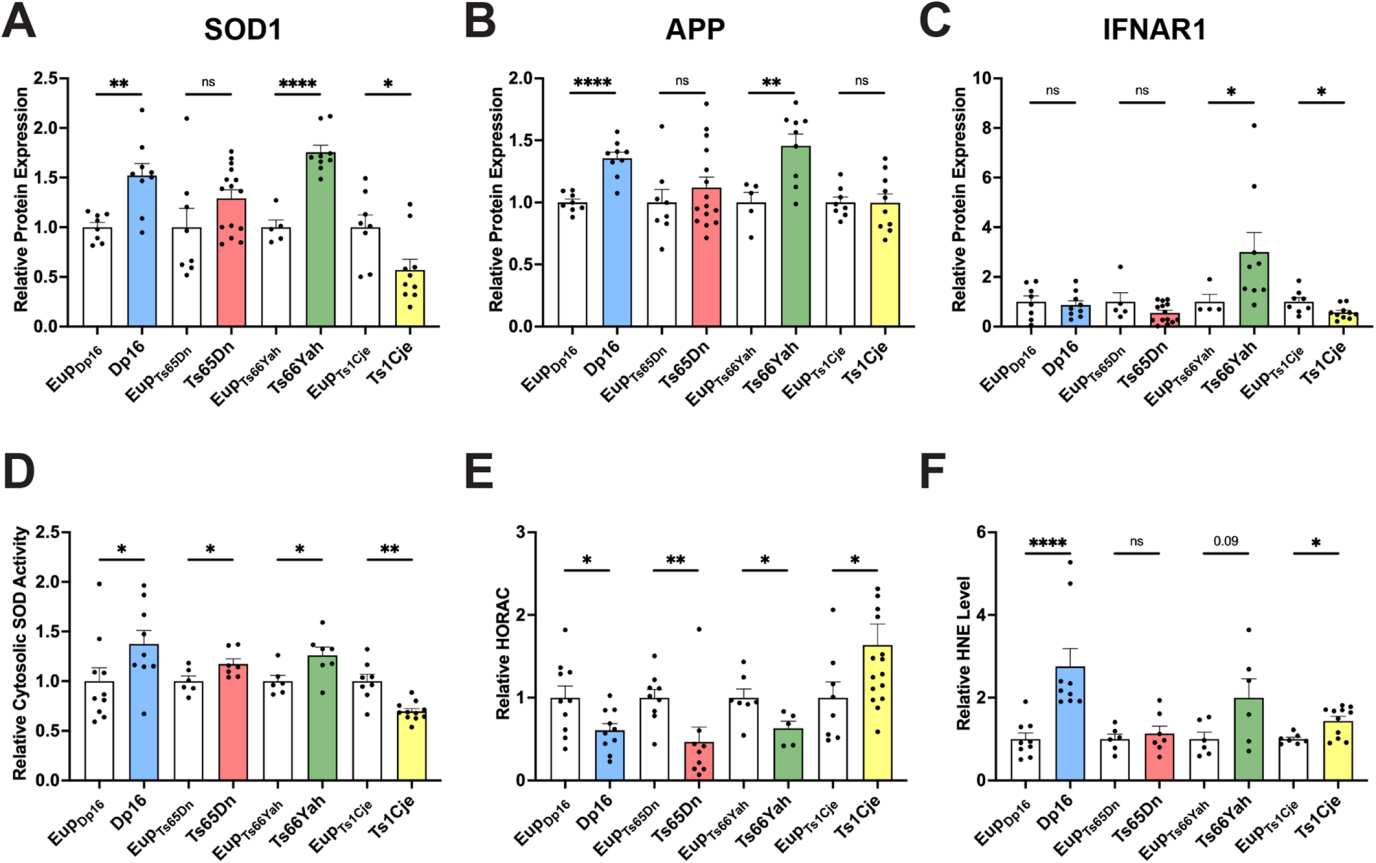
**Expression of SOD1, APP, and IFNAR1 and assessment of redox homeostasis in placentas of four mouse models of Down syndrome.** (A-C) Capillary western blotting was used to measure expression of SOD1 (A), APP (B), and IFNAR1 (C) in Dp16, Ts65Dn, Ts66Yah, and Ts1Cje placentas. Values shown are protein levels normalized to 40 ng of total protein in each sample. (D) Cytosolic SOD activity was significantly higher in trisomic placentas of Dp16, Ts65Dn, and Ts66Yah and was significantly lower in trisomic Ts1Cje placentas. Sample numbers: Dp16=9 trisomic, 10 euploid; Ts65Dn=7 trisomic, 6 euploid; Ts66Yah=7 trisomic, 6 euploid; Ts1Cje=11 trisomic, 8 euploid. (E) Hydroxy radical antioxidant capacity (HORAC) was lower in trisomic Dp16, Ts65Dn, and Ts66Yah placentas and higher in trisomic Ts1Cje placentas. Sample numbers: Dp16=10 trisomic, 10 euploid; Ts65Dn=9 trisomic, 9 euploid; Ts66Yah=5 trisomic, 7 euploid; Ts1Cje=16 trisomic, 8 euploid. (F) 4-hydroxynonenal (HNE) levels were significantly increased in Dp16 and Ts1Cje trisomic placentas; Ts66Yah increased HNE levels approached significance. Sample numbers: Dp16=9 trisomic, 9 euploid; Ts65Dn=7 trisomic, 6 euploid; Ts66Yah=6 trisomic, 6 euploid; Ts1Cje=11 trisomic, 7 euploid. For D-F, each sample was tested in triplicate, and the average for each sample is plotted and was used for statistical analyses. Error bars=s.e.m. Unpaired, two-tailed *t*-tests were performed for datasets with normal distribution, and two-tailed Mann–Whitney tests for datasets with non-normal distribution. ns, not significant; *P<0.05, **P<0.01, ****P<0.0001.

Given the significantly altered levels of *Sod1*, *App*, and *Ifnar1* RNA, we examined expression of the proteins encoded by these three genes by capillary western blotting. SOD1 showed significantly higher expression in trisomic Dp16 and Ts66Yah placentas (1.52- and 1.76-fold increase relative to euploid, respectively; Fig. 4A). In contrast, SOD1 in trisomic Ts1Cje placentas was reduced (relative protein level of 0.57 of euploid), corresponding to the trend toward lower full-length *Sod1* RNA levels (Fig. 4A, Fig. 1D; Fig. S1D). Increased APP expression was apparent in trisomic Dp16 and Ts66Yah placentas (1.36- and 1.46-fold increase relative to euploid, respectively), while Ts65Dn and Ts1Cje trisomic placentas showed no differences in APP expression compared to euploid controls (Fig. 4B). IFNAR1 expression showed variability in both euploid and trisomic placentas (Fig. 4C). A threefold increase in IFNAR1 expression was apparent in Ts66Yah trisomic placentas, and IFNAR1 expression was decreased in Ts1Cje trisomic placentas (relative protein level of 0.57 of euploid; Fig. 4C). Overall, trisomy-induced changes in RNA and protein levels were concordant for *Sod1*/SOD1 and *App*/APP in Dp16, Ts66Yah, and Ts1Cje, and for *Ifnar1*/IFNAR1 in Ts66Yah.

### Functional assays for SOD1 and oxidative stress

Several trisomic genes from *Hsa*21 and the syntenic *Mmu*16 region may alter redox homeostasis in DS and in mouse models of DS, including *Sod1* and *App* (Barone et al., 2018; Lott et al., 2006). To examine SOD1-related changes, cytosolic SOD activity was assessed in placental lysates, as SOD1 shows primarily cytosolic localization (Chang et al., 1988; Crapo et al., 1992; Okado-Matsumoto and Fridovich, 2001). Cytosolic SOD activity was increased in Dp16, Ts65Dn, and Ts66Yah placentas and decreased in trisomic Ts1Cje placentas (Fig. 4D), directly correlating with the altered SOD1 protein levels in each model (Fig. 4A). None of the models showed any changes in mitochondrial SOD activity (which primarily measures SOD2 activity) or catalase activity, which acts downstream to remove H_2_O_2_ generated by SOD1 activity (Fig. S4A,B). Since cytosolic SOD1 activity was elevated in Dp16 and Ts66Yah, unchanged catalase levels indicate an altered ratio of SOD1 and catalase activity in these two models, which could lead to excess H_2_O_2_ and subsequent oxidative stress.

Hydroxyl radical antioxidant capacity (HORAC) and total antioxidant capacity (TAC) assays were performed to measure placental antioxidant activity. Dp16, Ts65Dn, and Ts66Yah placentas all showed significantly lower HORAC levels (Fig. 4E) and trended towards decreasing TAC levels in trisomic placentas (Fig. S4C), suggesting these three models show a reduced ability to mitigate oxidative stress. Conversely, Ts1Cje mice showed higher HORAC and TAC levels in trisomic placentas (Fig. 4E; Fig. S4C). In addition, increases in 4-hydroxynonenal (HNE) levels, which measure lipid peroxidation, were observed in Dp16, Ts66Yah, and Ts1Cje trisomic placentas, with those in Dp16 and Ts1Cje achieving statistical significance (Fig. 4F).

No elevation in relative protein carbonyl level, which measures general oxidative damage to proteins, was observed in any of the models (Fig. S4D). In addition, β-galactosidase activity levels were either reduced (in Ts65Dn) or unchanged (Fig. S4E), suggesting no increase in cellular senescence as a result of trisomy.

### Assays for APP metabolism

Increased APP levels in DS are associated with unbalanced proteolytic processing of APP that leads to a larger pool of Aβ fragments, aggregation of Aβ fragments, increased levels of amyloid beta (Aβ)-42, and increased production of reactive oxygen species (Barone et al., 2018; Butterfield et al., 2001; Head and Lott, 2004). Although APP expression was elevated in Dp16 and Ts66Yah trisomic placentas (Fig. 4B), no significant differences were seen in Aβ-40 and Aβ-42 levels between trisomic and euploid placentas (Table S7A). Of note, a trend towards increased Aβ-42 was present in trisomic Ts66Yah placentas, in particular in placentas associated with trisomic embryos classified as a severe phenotypic group (Ts66Yah_severe_), based on more extensive embryonic brain and body weight reduction (Adams et al., 2024) (Table S7B).

### Inflammatory factor expression

IPA and Metascape analyses identified enriched pathways related to immune regulation or inflammation in Dp16, Ts65Dn, and Ts66Yah trisomic placentas. Therefore, immunoassays for cytokines, chemokines, and proteins related to STAT signaling and NF-κВ pathways were performed using placental protein lysates from all four models. No global changes in these inflammatory factors were observed, but individual factors were altered in trisomic placentas relative to euploid placentas, as follows: upregulation of IL-4 in Ts1Cje and downregulation of IFN-γ in Ts66Yah, IL-1β in Dp16, IL-5 in Dp16 and Ts65Dn, IL-6 in Ts65Dn, and c-Myc in Ts65Dn (Table S8).

### Immunofluorescent detection of SOD1 and APP in euploid and Ts66Yah placentas

We previously showed that trisomic Dp16, Ts65Dn, Ts66Yah, and Ts1Cje embryos can be statistically classified into mild and severe groups based on the extent of embryonic brain and body weight reduction (Adams et al., 2024). This study also showed that Ts66Yah exhibited the largest reduction in placental volume (Adams et al., 2024). Therefore, we used immunofluorescence to examine labyrinth structure and the expression of APP, SOD1, IFNAR1, and IFNAR2 in six euploid and 11 trisomic placentas (six Ts66Yah_mild_, five Ts66Yah_severe_).

The embryonically derived portion of the mouse placenta consists of two regions: the junctional zone (JZ), which provides hormones and growth factors, and the labyrinth, which governs maternal-fetal exchange. In the labyrinth, fetal and maternal blood are separated by a three-layered membrane consisting of sTGCs, the SynT-I layer, and the SynT-II layer. We examined labyrinth structure using immunofluorescence for monocarboxylate transporter 1 and 4 (MCT1 and MCT4), markers for SynT-I and SynT-II, respectively, and platelet/endothelial cell adhesion molecule 1 (PECAM1/CD31), a marker for vascular endothelial cells in the labyrinth. No gross structural differences in the labyrinth or differences in PECAM1, MCT1, or MCT4 levels were observed between euploid and trisomic Ts66Yah placentas, although MCT4 showed a trend towards reduced expression in trisomic placentas (Fig. S5A).

**Fig. 5.**
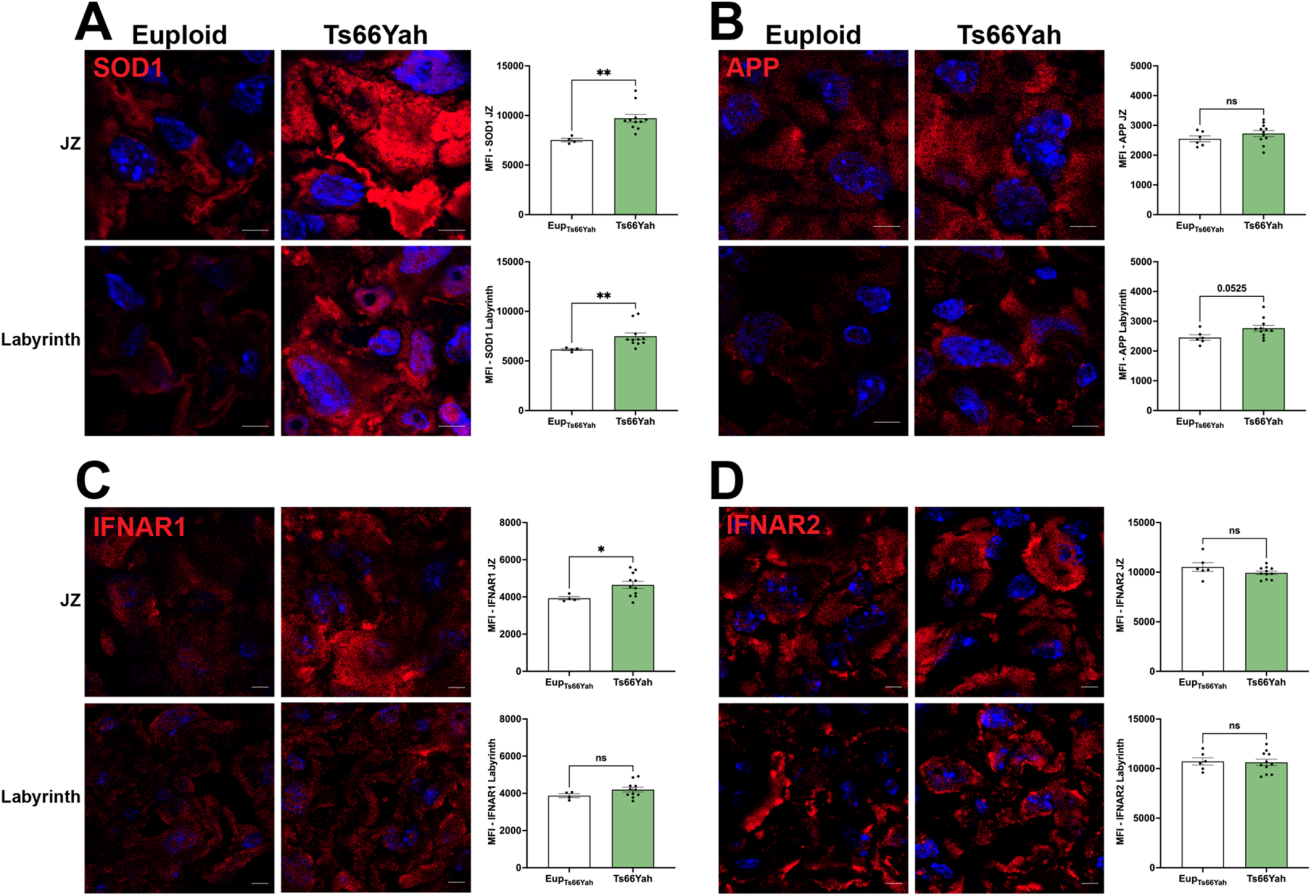
**Distribution of SOD1, APP, IFNAR1, and IFNAR2 protein in Ts66Yah placentas.** (A-D) Representative images of immunofluorescence staining are shown for SOD1 (A), APP (B), IFNAR1 (C), and IFNAR2 (D). For each panel, junctional zone (JZ) regions are shown at the top, and labyrinth at the bottom. Red=SOD1, APP, IFNAR1, or IFNAR2 and blue=Hoechst staining of nuclei. Graphs show the mean fluorescence intensity (MFI) of each target protein in each placental subregion. Sample numbers were: Eup *N*=6, trisomic *N*=11, with 6 Ts66Yah_mild_ and 5 Ts66Yah_severe_ trisomic placentas. Error bars=s.e.m. Unpaired, two-tailed *t*-tests were performed for datasets with normal distribution, and two-tailed Mann–Whitney tests for datasets with non-normal distribution. ns, not significant; *P<0.05; **P<0.01. Scale bars: 5 µm.

SOD1 expression in euploid and trisomic placentas was apparent throughout the placenta, with strong SOD1 expression in trophoblast giant cells (TGCs) of the junctional zone (JZ), and moderate expression in the labyrinth. Quantification of SOD1 showed significantly higher expression in both the JZ and the labyrinth of trisomic Ts66Yah placentas relative to euploid (Fig. 5A). Furthermore, SOD1 expression in the JZ and the labyrinth varied according to embryonic phenotype severity, with Ts66Yah_severe_ placentas showing higher SOD1 expression in both regions (Fig. S5B).

APP was expressed in the JZ and the labyrinth in both genotypes. Quantification showed increased APP expression in the trisomic labyrinth that approached significance (*P*=0.0525) but no difference in APP expression in the trisomic JZ (Fig. 5B). No differences in APP expression were present between Ts66Yah_mild_ and Ts66Yah_severe_ placentas in either structure (Fig. S5B).

Expression of IFNAR1 and IFNAR2 was visible in the JZ and labyrinth in both euploid and trisomic placentas. IFNAR1 exhibited significantly increased expression in the JZ of trisomic placentas (*P*=0.046), and a trend towards increased expression in the labyrinth (*P*=0.171) (Fig. 5C; Fig. S5B). Interestingly, Ts66Yah_mild_ placentas showed higher IFNAR1 expression than euploid and Ts66Yah_severe_ in both the JZ and the labyrinth (Fig. S5B). *Ifnar1* expression in Ts66Yah measured by qPCR showed a corresponding trend towards higher levels of expression in Ts66Yah_mild_ placentas, but these differences did not reach statistical significance (data not shown). In contrast, no differences were detected in IFNAR2 expression between euploid and Ts66Yah trisomic placentas in either the JZ or the labyrinth (Fig. 5D; Fig. S5B).

In summary, these results suggest that trisomy for the *Hsa*21 orthologs at dosage imbalance in Ts66Yah causes upregulation of SOD1 in the JZ and labyrinth, upregulation of APP in the labyrinth, and upregulation of IFNAR1 in the JZ. Placental expression of SOD1 and IFNAR1 varies with embryonic phenotypic severity, with higher SOD1 expression associated with the trisomic Ts66Yah_severe_ phenotype, and higher IFNAR1 expression in both the JZ and labyrinth associated with the trisomic Ts66Yah_mild_ phenotype.

## DISCUSSION

Limited research exists on placental gene expression in human fetuses with T21 or mouse models of DS (Bianco et al., 2016; Kipp et al., 2012; Pennings et al., 2011; Rozovski et al., 2007; Wong et al., 2018). To address this knowledge gap, we identified placental transcriptomic differences and downstream proteomic and functional changes that are present during late gestation in four mouse models of DS, to better understand the effects of trisomy for *Hsa*21 orthologs on this essential organ. Our comparison of four models permitted identification of similarities in gene dysregulation, revealing common trisomy-induced alterations, along with recognition of differences, suggesting unique features that may be helpful guides for choosing mouse models in future developmental studies.

The shared DEGs and MDGs identified in this study revealed a core set of upregulated *Hsa*21 orthologs that may alter placental function. In addition, the MDGs with previously identified effects on placental development, structure or function as well as cardiac, neuronal, or lethal phenotypes (Table S3) are intriguing. Emerging evidence showing direct effects of the placenta on heart development (Adams et al., 2000; Barak et al., 1999; Maruyama et al., 2016; Radford et al., 2023; Torregrosa-Carrion et al., 2021) suggests these MDGs may be relevant to embryonic phenotypes. For example, *Gpc3*, *Mecom*, and *Vcan* are each associated with structurally abnormal cardiac phenotypes, including ventricular septal defects (https://www.informatics.jax.org/). This is notable because congenital heart defects are present in ∼50% of individuals with DS, and the majority of these are septal defects (Dimopoulos et al., 2023).

Trisomic placentas from all four models showed altered pathways related to the extracellular network, detection of extracellular/sensory stimulus, and immune system-related pathways, suggesting these may be common alterations regardless of the genomic differences in each model. These results paralleled previous studies that showed developmental alterations in extracellular matrix or immune pathways as a result of trisomy in Dp16 heart, lymph nodes, brain and embryonic mesenchyme as well as in human blood, lymphoblastoid cell lines, primary circulating monocytes and T cells, fibroblasts, induced pluripotent stem cell (iPSC)-derived cardiac cells, and iPSC-derived differentiating neuroectoderm (Chi et al., 2023; Martinez et al., 2024; Mollo et al., 2022; Sullivan et al., 2016; Waugh et al., 2023). In addition, altered sensory stimulus pathways may be relevant to developmental changes in preparation for postnatal life, as late gestation human fetal transcripts exhibit upregulation of genes related to sensory perception (Maron et al., 2007).

The transcriptional and proteomic differences present among the four models may be caused by the unique sets of trisomic genes in each model. These differences may also be attributable to chromosomal number, as Ts65Dn and Ts66Yah are aneuploid models, while Dp16 and Ts1Cje are diploid. This is supported by a recent study that demonstrated that the presence of an extra chromosome in mouse models of DS strongly affects gene expression changes and behavioral phenotypes in comparison to those in diploid models (Xing et al., 2023). For Ts65Dn, gene expression differences relative to the other models could also stem from the fact that the pregnant dam was trisomic. Therefore, the RNA of both euploid and trisomic Ts65Dn placentas contained transcripts from trisomic maternal cells, which may have lessened the sensitivity of bulk gene expression analysis.

Ts1Cje showed distinct transcriptomic and proteomic differences from the other three models. This could be due to specific effects from the *Mmu*12 monosomic genes, general effects of having approximately half the number of trisomic genes, or specific effects from the ∼60-70 *Mmu*16 genes that are trisomic in the other models but disomic in Ts1Cje, such as *Sod1*, *App*, *Atp5j*, *Cct8*, *Usp16*, *Gabpa*, and *Ltn1*. Downregulation of SOD1/SOD activity in Ts1Cje suggests that the abnormal *Sod1* transcript in Ts1Cje may initiate regulatory feedback mechanisms that downregulate SOD1 in the placenta. SOD1 downregulation does not occur in all Ts1Cje tissues, as Ts1Cje mice have been reported to show normal SOD enzymatic activity (Sago et al., 1998), suggesting tissue-specific or developmental differences in *Sod1* expression.

Overall, these studies alongside previous analyses suggest that Ts66Yah may be an excellent model for *in vivo* placental/embryonic studies. Ts66Yah is aneuploid, is only trisomic for *Hsa*21 orthologs, and shows more congenital heart defects, renal pelvis dilation, and reductions in placental size compared to the other three models (Adams et al., 2024; Duchon et al., 2022; Guedj et al., 2023). In this study, Ts66Yah was the only model with consistent increases in RNA/protein expression for *Sod1/*SOD1, *App/*APP, and *Ifnar1/*IFNAR1 alongside alterations in redox homeostasis.

### SOD1, APP, IFNAR1, and IFNAR2 expression in the placenta

Dp16, Ts65Dn, and Ts66Yah showed placental overexpression of *App*, *Sod1*, and *Ifnar1/2* to varying degrees. This overexpression may contribute to altered placental development or function, and thus these genes may be relevant targets for DS therapy. For example, data exist that link pathways governed by these genes to placental syncytialization, which shows abnormalities in T21 (detailed below). Dysregulation of these genes may contribute to trisomy-associated growth reduction, suggested by elevated SOD1 expression in Ts66Yah_severe_ placentas and elevated IFNAR1 expression in Ts66Yah_mild_ placentas (Fig. S5B). The intriguing finding of upregulated IFNAR1 protein in Ts66Yah_mild_ placentas will need confirmation in follow-up comparative studies of Ts66Yah_mild_ and Ts66Yah_severe_ placentas, but it may suggest notable variability in IFN pathway activation exists that may associate with body size. Future studies at the single-cell level will be needed to more precisely understand the developmental roles of these genes and how their dosage imbalance may contribute to placental abnormalities.

Most APP-related studies in DS have focused on adult neurological effects rather than developmental effects of *APP* dosage imbalance, because elevated APP expression contributes to Alzheimer's disease neuropathology and accumulation of Aβ deposits in individuals with DS (Wiseman et al., 2015). However, APP also influences multiple aspects of neurodevelopment, including migration, proliferation, and differentiation of neural progenitors as well as synaptogenesis (Coronel et al., 2019; van der Kant and Goldstein, 2015). In addition, APP is highly expressed in the human placenta, and aggregates of APP proteolytic fragments have been identified in placentas in pre-eclampsia, suggesting important roles in placental development and function (Buhimschi et al., 2014; Cheng et al., 2016). APP is overexpressed in human T21 placentas, and *in vitro* overexpression of APP in trophoblasts increases apoptosis and decreases cell growth and migration (Wong et al., 2018). Furthermore, APP overexpression in BeWo choriocarcinoma cells reduces cell syncytialization/fusion (Wong et al., 2018), suggesting that APP overexpression may contribute to reduced syncytialization that has been observed in histological sections of T21 placentas and *in vitro* T21 trophoblast cultures (Frendo et al., 2000; Pidoux et al., 2004; Roberts et al., 2000).

Elevated placental expression of *App*/APP mRNA and protein was present in Dp16 and Ts66Yah, and APP expression approached significance by immunofluorescence in the Ts66Yah labyrinth, corresponding with elevated APP expression in labyrinth sTGCs from single-nuclei RNA-Seq analyses (Marsh and Blelloch, 2020). While these results suggest the labyrinth is a primary site of APP expression and function, follow-up studies with multiple developmental timepoints will be needed to fully ascertain the effects of elevated *App* expression on the trisomic placenta.

This study showed reduced antioxidant capacity in Dp16, Ts65Dn, and Ts66Yah placentas and increased lipid peroxidation in Dp16 and Ts66Yah placentas, suggesting trisomy-associated increases in oxidative stress. Elevated lipid peroxidation correlates with previous studies showing that increased expression of wild-type SOD1 leads to greater lipid peroxidation levels (Lee et al., 2001). Several studies suggest oxidative stress is increased in individuals with DS during prenatal development (Buczyńska et al., 2021; Busciglio and Yankner, 1995; Perluigi and Butterfield, 2012; Slonim et al., 2009). While multiple genes that are trisomic in DS may be contributing to these changes, including *App*, *Bace2*, *Bach1*, and *Sod1* (Barone et al., 2018; Head and Lott, 2004; Perluigi and Butterfield, 2012), *Sod1* has been a focus of T21 studies related to oxidative stress due to its primary role in antioxidant defense. Any perturbation of *Sod1* expression may be detrimental to development, as female *Sod1* knockout mice show reduced fertility due to post-implantation embryonic lethality (Ho et al., 1998), and ∼2-fold overexpression of SOD1 *in vitro* results in reduced cellular proliferation and increased lipid peroxidation (Lee et al., 2001). The detrimental effects of *Sod1* overexpression may be SOD1's production of the reactive oxygen species H_2_O_2_ in the process of detoxifying superoxide. This H_2_O_2_ is normally inactivated by glutathione peroxidase, catalase, or the thioredoxin system, but if increased SOD1 levels occur without concomitant increase of downstream antioxidants, then elevated H_2_O_2_ persists and leads to oxidative stress (Barone et al., 2018).

SOD1 is expressed in the euploid placenta, and oxidative stress/elevated reactive oxygen species (ROS) are normal placental features that fluctuate as development progresses and may regulate stages of differentiation (Myatt and Cui, 2004). Trophoblast syncytialization is sensitive to SOD1 levels, as *in vitro* overexpression of SOD1 in euploid cytotrophoblasts inhibits hormone production and blocks cell fusion, and high SOD1 expression and activity are present in trophoblast cells isolated from T21 placentas (Frendo et al., 2001; Pidoux et al., 2004). Increased SOD1 expression has also been described in proteomic and immunohistochemistry analyses of mid-gestation T21 placentas (Sun et al., 2011). The striking upregulation of SOD1 in the JZ and labyrinth of Ts66Yah placentas suggests that SOD1 pathways and redox balance may be altered in trisomic placentas and warrants further investigation.

Recent studies have shown that activation of interferon (IFN) signaling pathways in multiple cell types are relevant to DS phenotypes (Chi et al., 2023; Galbraith et al., 2023; Sullivan et al., 2016; Waugh et al., 2023). Correction of the trisomic IFN receptor cluster to diploid levels in Dp16 mice rescued congenital heart defects, craniofacial alterations, and cognitive impairments (Waugh et al., 2023). JAK inhibition, which targets downstream effects of IFN activation, also showed phenotypic improvement in mouse models of DS (Chi et al., 2023; Galbraith et al., 2023; Tuttle et al., 2020), and a clinical trial examining the JAK inhibitor tofacitinib in individuals with DS showed promise for alleviating autoimmune-related skin conditions (Rachubinski et al., 2024). Therefore, elevated IFN pathways may be important therapeutic targets in DS. This study showed that immune system/inflammation pathways were upregulated in Dp16 and Ts66Yah placentas, suggesting the validity of these two models for testing if activated IFN signaling alters placental development and function and if IFN pathway inhibition can improve placental phenotypes. While the presence and effects of upregulated IFN signaling pathways in the T21 placenta remain to be elucidated, upregulation of IFN-induced transmembrane proteins impairs cytotrophoblast invasion, inhibits syncytiotrophoblast formation, and can lead to placental abnormalities and fetal resorption (Buchrieser et al., 2019; Degrelle et al., 2023; Zani et al., 2019). This demonstrates that two distinct placental developmental stages are regulated by IFN pathways.

### Relevance to prenatal development in DS

Human placental abnormalities can have detrimental effects, including fetal growth restriction, preeclampsia, and chronic disease susceptibility (Aplin et al., 2020; Brosens et al., 2011; Burton et al., 2016). Placental abnormalities associated with T21 may affect fetal development, making the placenta a potential therapeutic target to improve developmental outcomes for affected individuals. Prenatal clinical intervention is feasible due to the widespread availability of prenatal screening and diagnosis of T21 (Guedj and Bianchi, 2013), which would permit early treatment during critical brain developmental stages that occur *in utero*. To date, postnatal therapies to improve neurocognition in DS have largely been unsuccessful, and extensive analyses of model systems at different developmental timepoints are needed to determine the potential for prenatal therapies (Lee et al., 2020; Stagni and Bartesaghi, 2022). Mouse models will be informative tools to understand the effects of trisomy for *Hsa*21 orthologs on embryonic and placental development and function as well as pre-clinical testing of candidate therapeutic molecules.

### Limitations

This study had several limitations. Sample sizes were relatively small in the context of the phenotypic and transcriptomic variability that has been observed in T21 (Araya et al., 2022; Karmiloff-Smith et al., 2016; Lee et al., 2025; Thomas et al., 2020). The study examined a single timepoint (E18.5); a developmental time course will be more informative in future studies. Additionally, the gestational time of mice (∼21 days) is shorter than that of humans (40 weeks) and thus may not model time-dependent T21 placental phenotypes.

## MATERIALS AND METHODS

### Mouse breeding

Animal housing and breeding was carried out in a specific-pathogen-free facility in accordance with protocols approved by the Institutional Animal Care and Use Committee of the National Human Genome Research Institute (NHGRI) at the National Institutes of Health (NIH). Mice for three of the lines were purchased from The Jackson Laboratory (JAX; Bar Harbor, ME, USA): B6.129S7-Dp(16Lipi-Zbtb21)1Yey/J [abbreviated Dp(16)1/Yey or Dp16], 013530; B6.Cg-T(12;16)1Cje/CjeDnJ (abbreviated Ts1Cje), 004838; and B6EiC3Sn.- BLiA-Ts(1716)65Dn/DnJ (abbreviated Ts65Dn), 005252. For the Ts66Yah line, trisomic females were obtained from the Laboratory of Yann Herault (Université de Strasbourg, Centre National de la Recherche Scientifique), and were used to establish a Ts66Yah colony at the NIH (Guedj et al., 2023). Ts65Dn and Ts66Yah lines were maintained by crossing to B6EiC3Sn.BLiAF1/J F1 hybrid mice (003647, JAX) and Ts1Cje and Dp16 mice were maintained by breeding to C57Bl/6J mice (000664, JAX).

To obtain E18.5 embryos and associated placentas, trisomic Ts1Cje and Dp16 male mice were bred with C57BL/6J females. Trisomic Ts65Dn females were bred with B6EiC3Sn.BLiAF1/J males (due to male infertility), and trisomic Ts66Yah males (that retain fertility) were bred to B6EiC3Sn.BLiAF1/J females. Therefore, in this study the trisomic genetic material was only passed through the female in the Ts65Dn strain, while in the other three lines the trisomic genetic material was passed through the male, permitting embryonic development in a euploid uterine environment. Morphological differences of E18.5 embryos and placentas from these four crosses have been described previously (Adams et al., 2021, 2024).

### Placenta dissection

Matings were set up between 16:00 and 17:00 and separated the following morning. The presence of a vaginal plug was designated as E0.5. Pregnant females were euthanized following approved NHGRI animal care and use committee guidelines, and embryos and placentas were dissected at E18.5. Placental weights and diameters were measured immediately after dissection. All layers of the placenta were collected, including the decidua. The full placenta was halved, and then one half was cut in half again, yielding two quarters that were individually snap frozen in liquid nitrogen and stored at −80°C for RNA or protein extraction. The other placental half was fixed in 10% buffered formalin for 24 h and stored in 70% ethanol for histology.

### DNA and RNA extraction

DNA was extracted from tail biopsies using NucleoSpin XS kits (Macherey-Nagel, Bethlehem, PA). To determine trisomy and fetal sex, PCR was performed using the following primers and previously described amplification protocols. For Ts1Cje, F: CTCGCCAAAGGAATGCAAGGTCTGT and R: CCCTTGTTGAATACGCTTGAGGAGA (Olson et al., 2004). For Dp16, F: CTGCCAGCCACTCTAGCTCT and R: AATTTCTGTGGGGCAAAATG (Goodliffe et al., 2016). A positive control PCR reaction was used along with the trisomic-specific PCRs for both Ts1Cje and Dp16, F: AAGATCTGAGGCTCGCCAAG and R: CTTCGGGAGCAGGTACCCTA; (Goodliffe et al., 2016). For Ts65Dn, FMmu17: GACTTAGTAAGAGCAAGTGGC and RMmu16: AGGTAGAAAGATGTGAGGACAC and RMmu17: GGGCAACACTGGATCAATC (Duchon et al., 2011). For Ts66Yah, genotyping was initially performed using a simplex PCR, FMmu17: CTAGACTATAAACATGTCTCACTAC and RMmu16: GTATTCATTATGTGGAACTACCAGA, and follow-up PCR reactions to confirm genotypes were performed using a multiplex PCR, FMmu17: GGAAATATCGCACTTCACCAA and R-Ts66Yah CATGGGTCTGTGTGGTTTTCT and R-WT TCTAGGATCAGTGGGACTTTTGT (Duchon et al., 2022). Sex determination was performed by amplification of the male-specific gene *Sry*, F: GCTGGGATGCAGGTGGAAAA and R: TGATGGCATGTGGGTTCCTG (Aziz et al., 2018).

### MGI phenotype analyses

A batch search was performed at MGI (https://www.informatics.jax.org/batch) using MDGs compiled from all four models to identify genes with mammalian phenotype terms related to the placenta or embryonic/prenatal lethality.

### Microarray analysis and RT-qPCR

One-quarter of each placenta that contained all layers (including the decidua) was homogenized and used for RNA isolation. Total placental RNA was extracted using NuceloMag RNA/DNA extraction kits (Machery-Nagel). RNA was processed and hybridized on Mouse Clariom S HT 96-array plates (902970; Thermo Fisher Scientific, Waltham, MA, USA) using GeneChip WT Plus Reagent Kits following the manufacturer's protocol (902281; Thermo Fisher Scientific). Microarray data were analyzed using Transcriptome Analysis Console (TAC) software (version 4.0.0) (Thermo Fisher Scientific) using RMA normalization. Samples that failed pre-established, standard TAC quality control tests were excluded. Sample numbers were as follows: Dp16=20 trisomic, 20 euploid; Ts65Dn=19 trisomic, 17 euploid; Ts66Yah=9 trisomic, 14 euploid; Ts1Cje=24 trisomic, 17 euploid.

DEGs in trisomic placentas relative to euploid placentas were defined by FDR *P*-value <0.1 and fold change >|1.2| by ANOVA. Marginally dysregulated genes (MDGs) were defined by unadjusted *P*-value <0.05 and fold change >|1.2|, which yielded longer lists amenable to pathway analyses.

RT-qPCR was performed using a QuantStudio 7 Flex Real-Time PCR machine (Applied Biosystems/Thermo Fisher Scientific). Each qPCR reaction used 100 or 150 ng of cDNA synthesized from total placental RNA using SuperScript IV VILO cDNA synthesis kits (11756050, Thermo Fisher Scientific). Multiplex reactions with VIC and FAM labeling were performed in triplicate for each sample using TaqMan Multiple Master Mix (4461882, Thermo Fisher Scientific), intron-spanning TaqMan assays for each targeted gene, and TaqMan assays of either *Gapdh* or *Hprt* (Table S9). After removal of outlier wells, quantitation was performed using the delta-delta CT method. Significant changes in expression were identified by Student's *t*-test or Mann–Whitney test (if non-Gaussian distribution present), and gene expression changes were deemed significant if consistent alterations in expression were present in both *Gapdh* and *Hprt* control assays.

### Pathway analyses

Enriched pathway analyses were performed for placental MDGs of each model using Metascape (https://metascape.org) (Zhou et al., 2019). Metascape analysis clusters significantly enriched pathways together based upon functional similarity, and the top 20 significant functional clusters for each model were used for analyses. In addition, all pathways within the top 20 clusters for each model were manually re-categorized into nine biological groups: (1) Immune System, (2) Cell Stress/Cell Death/Defense Response, (3) Growth and Development, (4) Intracellular Network, (5) Extracellular Network, (6) Molecular Transport, (7) Metabolism, (8) Neuron and Brain, (9) Circulatory System.

Enriched pathway analyses were also performed for the placental MDGs of every model using Ingenuity Pathway Analysis (Winter 2024 version, Qiagen, Germantown, MD, USA). For comparison of predicted upstream regulators among the 4 models, upstream regulators with *P*-values <0.001 were used.

### Protein extraction and protein assays

One-quarter of each mouse placenta was homogenized in 300 µl of ice cold lysis buffer that was specific for each assay, as follows: SOD and catalase activity assays, 20 mM HEPES buffer (pH 7.2) containing 1 mM EGTA, 210 mM mannitol, 70 mM sucrose, and Halt protease and phosphatase inhibitor cocktail (78444, Thermo Fisher Scientific); TAC, HORAC, and β-galactosidase assays, 5 mM potassium phosphate (pH 7.4) containing 0.9% sodium chloride, 0.1% glucose, and Roche cOmplete™, Mini, EDTA-free Protease Inhibitor Cocktail (4693159001, Millipore Sigma); Luminex assays and capillary western blotting, MilliplexMAP Cell Signaling Universal Lysis Buffer (43-040, Millipore Sigma) and Halt protease and phosphatase inhibitor cocktail. Lysates were centrifuged at 10,000 ***g*** for 15 min at 4°C, and the supernatant was collected for protein assays. For SOD activity assays, lysates were centrifuged at 1500 ***g*** for 5 min at 4°C, the supernatant was collected and centrifuged at 10,000 ***g*** for 15 min, and the resulting supernatant was collected for cytosolic SOD assays while the pellet was re-suspended in 30 µl of lysis buffer for mitochondrial SOD assays. All samples were stored at −80°C until usage in assays. Protein concentrations were detected using the Pierce™ BCA Protein Assay Kit (23227, Thermo Fisher Scientific).

All samples were assessed in triplicate, and absorbance and fluorescence measurements were obtained using a Cytation 5 Cell Imaging Multimode Reader (Agilent Technologies, Santa Clara, CA, USA). Assay kits were used following the manufacturer's instructions, as follows: Superoxide Dismutase Assay Kit (706002; Cayman Chemical, Ann Arbor, MI, USA), Catalase Assay Kit (Cayman Chemical, 707002), Antioxidant Assay Kit (709001, Cayman Chemical), OxiSelect™ Hydroxyl Radical Antioxidant Capacity (HORAC) Activity Assay kit (STA-346; Cell Biolabs, Inc., San Diego, CA, USA), OxiSelect™ HNE Adduct Competitive ELISA Kit (STA-838; Cell Biolabs, Inc.), OxiSelect™ Protein Carbonyl ELISA Kit (STA-310; Cell Biolabs, Inc.), and 96-Well Cellular Senescence Activity Assay Kit (CBA-231, Cell Biolabs, Inc.).

### Capillary western blotting

Expression of SOD1, APP, and IFNAR1 was measured using a Simple Western capillary electrophoresis system (R&D Systems) and a Peggy Sue Simple Western automated capillary immunoassay system (ProteinSimple, Bio-Techne, Minneapolis, MN, USA) according to the manufacturer's instructions. Target protein measurements were normalized to 40 ng of total protein in each sample. Primary antibodies and concentrations are listed in Table S9, and secondary antibody was anti-rabbit Horseradish Peroxidase (042-206, ProteinSimple, Bio-Techne). Sample numbers were as follows: Dp16=9 trisomic, 8 euploid; Ts65Dn=15 trisomic, 8 euploid; Ts66Yah=9 trisomic, 5 euploid; Ts1Cje=10 trisomic, 8 euploid.

### Luminex assays

Luminex bead-based assays, which quantify multiple analytes simultaneously, were performed using a Luminex 200 Analyzer with xPONENT for LX100/LX200 software (MilliporeSigma, Burlington, MA, USA). Euploid and trisomic placenta protein lysates from all four models were examined using Luminex kits related to the immune system or inflammation. The protein amounts and kits used were as follows: 80 ng/well for the MILLIPLEX^®^ Mouse High Sensitivity T Cell Magnetic Bead Panel (MHSTCMAG-70K, Millipore Sigma); 20 ng/well for the MILLIPLEX^®^ MAP STAT Cell Signaling Magnetic Bead 5-Plex Kit - Cell Signaling Multiplex Assay (48-610MAG, Millipore Sigma); 20 ng/well for the MILLIPLEX^®^ NF-κB Signaling Magnetic Bead Kit 6-plex Kit - Cell Signaling Multiplex Assay (48-630MAG, Millipore Sigma); and 50 ng/well for Mouse Amyloid Beta Magnetic Bead Panel 96-Well Plate Assay (MABMAG-83K, Millipore Sigma). Plate preparations were performed following the manufacturer's instructions, and all placental lysates and control samples were assessed in duplicate. Quantification using a standard curve or calculation of mean fluorescence intensity was performed with Belysa Immunoassay Curve Fitting Software (version 1.2.1, MilliporeSigma). Duplicate sample means were calculated for each factor in each placenta lysate and used for statistical analysis.

### Immunofluorescence

For immunofluorescence staining of Ts66Yah placentas, formalin-fixed placental halves were paraffin embedded, cut along the plane of the halved tissue into 4-6 µm sections, and mounted onto slides (Histoserv, Inc., Germantown, MD, USA). Sample numbers were as follows: Eup *N*=6, trisomic *N*=11, with six mild and five severe trisomic placentas. Unstained slides were baked at 58-60°C for 1 h and then deparaffinized. Antigen retrieval was performed in a 2100 Retriever pressure cooker (62700-10, Electron Microscopy Sciences, Hatfield, PA, USA) with R-Buffer A (62706-10; Electron Microscopy Sciences). Tissue sections were then soaked with freshly-prepared 0.05% KMnO_4_ solution for 10 min at room temperature (R/T) to reduce non-specific staining of red blood cells. After washing twice with 1× PBS, both blocking and cellular permeabilization were performed with Carrier Solution (PBS with 0.5% bovine serum albumin, and 0.3% Triton X), and then sections were incubated with primary antibody (Table S9) diluted with Carrier Solution overnight at 4°C with shaking. After washing (1×10 min with Carrier Solution, then 2×10 min with 1× PBS), slides were incubated with secondary antibodies (Table S9) diluted with Carrier Solution for 1 h at R/T with shaking. After washing with Carrier Solution and PBS, Hoechst staining (15 µg/ml) was performed for 10 min at R/T. After washing for 10 min in PBS and rinsing in dH2O, coverslips were mounted using Prolong Gold Antifade Reagent (P36934, Invitrogen/Thermo Fisher Scientific).

Fluorescent imaging was performed with a Zeiss LSM 900 with Airyscan 2 detector and ZEN (blue edition) software (v3.8, Carl Zeiss Co., Oberkochen, Germany). Two sections were analyzed for every placenta. For each section, one tiling image spanning all three placental subareas (decidua, JZ, and labyrinth) and three individual labyrinth images (1024 pixels×1024 pixels) were generated at 63× (APP, SOD1, MCT1, MCT4) or 40× (IFNAR1, IFNAR2) magnification using identical laser settings. For JZ measurements, three individual images (500 pixels×1350 pixels) were generated from each tiling image.

### Quantitative analysis of immunofluorescence images

Image segmentation was performed using LabKit add-in software in ImageJ-Fiji before measuring fluorescence intensities. In color merged images, foreground areas (the placental tissue) and background areas (inside the fetal and maternal vessels and maternal blood pool regions) were classified manually for each image, and then segmentation was used to define a masked image that solely measured placental tissue. Low cut-off values to compare target protein intensity between euploid and trisomic placentas were determined for each staining experiment by averaging the lower cut-off values for each euploid placenta image using the ImageJ OTSU automatic thresholding algorithm. Mean fluorescence intensity (MFI) was used to measure protein expression levels. The investigator was unaware of sample genotype for all stages of quantitative analysis.

### Statistical analysis

ANOVA analyses of microarray datasets were performed using TAC (Thermo Fisher Scientific, version 4.0.0). PCA was performed using Partek Genomics Suite Partek (Partek Incorporated, version 7.21.1119). All other statistical analyses were performed using GraphPad Prism (version 10.3.1). Normality was evaluated for each dataset, and appropriate parametric or nonparametric tests (Mann–Whitney or Kruskal–Wallis) were used to compare euploid and trisomic placentas or placentas from euploid, mild trisomic, and severe trisomic mice, as previously described (Adams et al., 2024). Sample sizes were maximized as much as possible given the limited availability of tissue samples: the minimum *n* for each test was predicted to identify ≥13% difference in means with 95% probability (*P*-value 0.05).

## Supplementary Material

10.1242/biolopen.062296_sup1 Supplementary information

Table S1. Differential gene expression analysis results for MDGs and all transcripts in the placenta of four mouse models of Down syndrome.

Table S3. (A) Mouse Genome Informatics query identified 279 placental MDGs in mouse models of Down syndrome that are associated with prenatal lethality, (B) Mouse Genome Informatics query identified 54 placental MDGs with previously identified placental phenotypes. (C) Placental Mammalian Phenotype Terms associated with the 54 MDGs with placental phenotypes. (D) A subset of the MDGs with placental mammalian phenotypes also have heart and brain phenotypes.

Table S4. Metascape enriched pathway analyses for placental MDGs from Dp16, Ts65Dn, Ts66Yah, and Ts1Cje.

Table S5. Enriched canonical pathways identified using Ingenuity Pathway Analysis for placental MDGs from Dp16, Ts65Dn, Ts66Yah, and Ts1Cje.
