# Supplementary material for: The placental effects of trisomy for human chromosome 21 orthologs in four mouse models of Down syndrome
Source: Biol Open. 2025 Dec 18;14(12):bio062296. doi: 10.1242/bio.062296 (PMC12755066; doi:10.1242/bio.062296)
Supplement: Supplementary information [file biolopen-14-062296-s1.pdf]

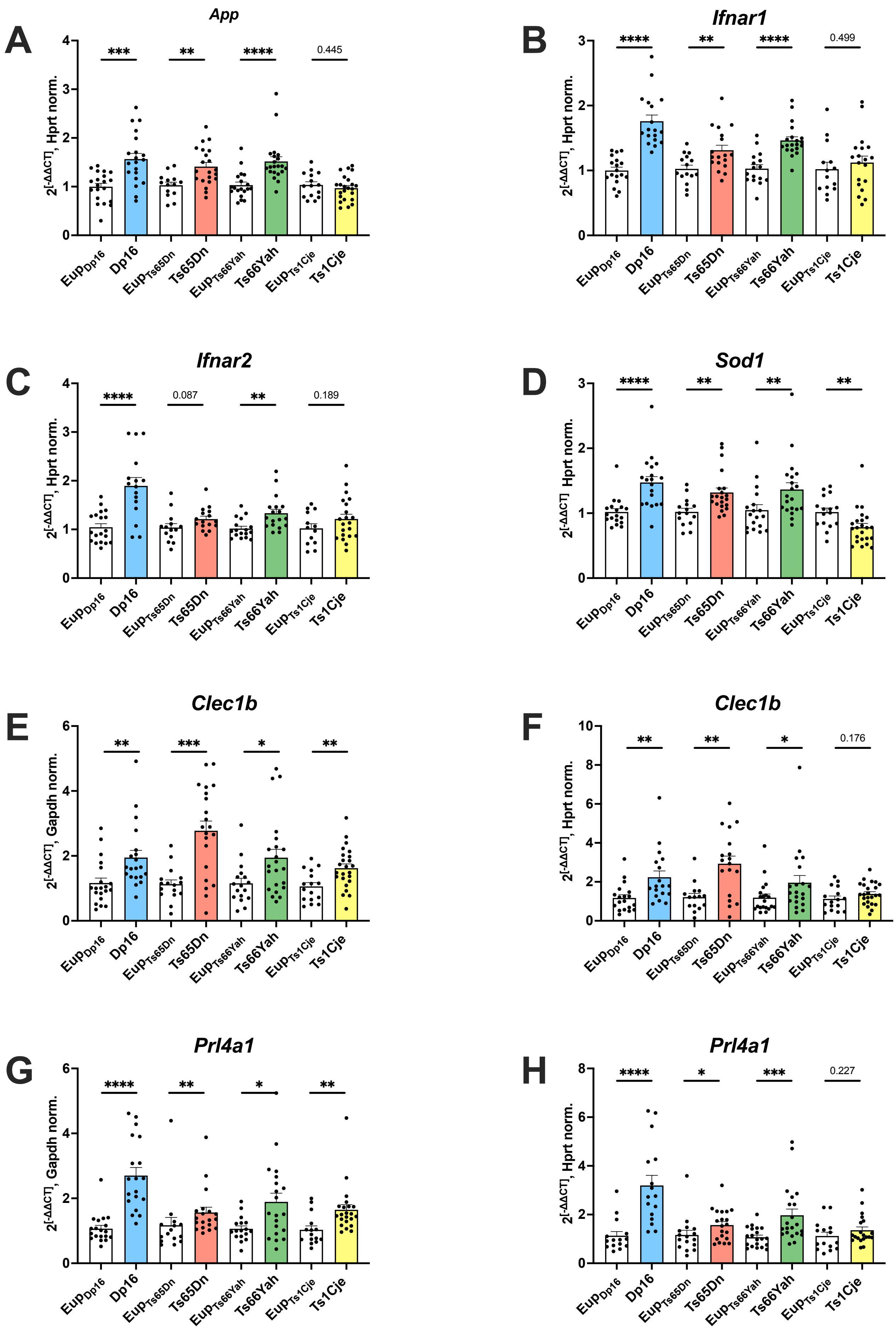

Biology Open • Supplementary information

Fig. S1. RT-qPCR validation of placental DEGs and MDGs. A) *App*, **B)** *Ifnar1*, **C)** *Ifnar2,* **D)** *Sod1,* **E-F)** *Clec1b,* and **G-H)** *Prl4a1* expression in placentas from Dp16, Ts65Dn, Ts66Yah, and Ts1Cje. A – D, F, and H are normalized with *Hprt*, and E and G are normalized with *Gapdh*. Sample numbers were: Dp16 = 23 trisomic, 22 euploid; Ts65Dn = 23 trisomic, 18 euploid; Ts66Yah = 22 trisomic, 21 euploid; Ts1Cje = 26 trisomic, 18 euploid. Error bars = SEM. Unpaired, two-tailed t tests were performed for datasets with normal distribution, and two-tailed Mann-Whitney tests for datasets with non-normal distribution. ns, not significant; * *p*-value < 0.05, ** *p*-value < 0.01, *** *p*-value < 0.001, **** *p*-value < 0.0001.

Biology Open • Supplementary information

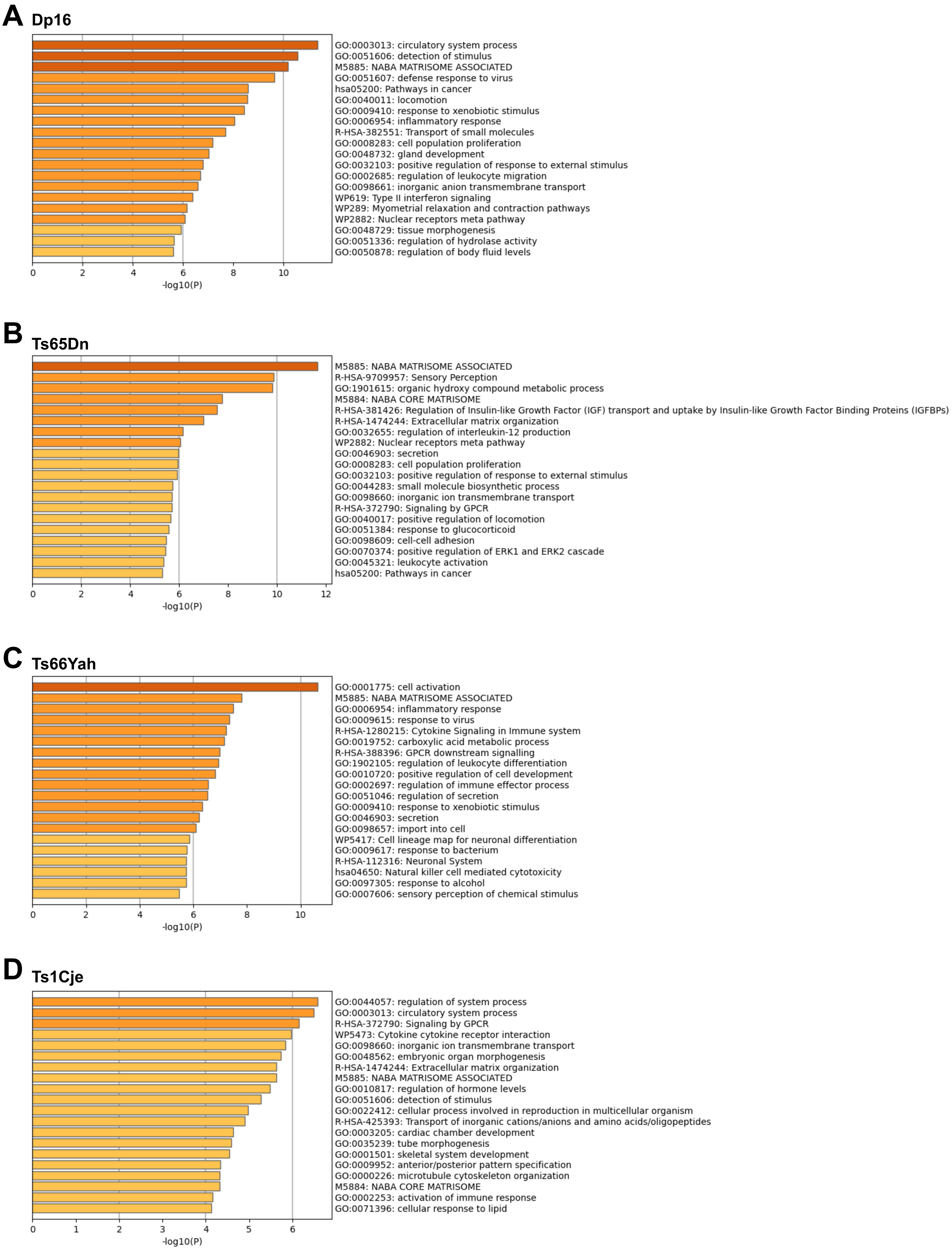

Fig. S2. Functional pathways clusters identified by Metascape enriched pathway analyses for placental MDGs in Dp16, Ts65Dn, Ts66Yah, and Ts1Cje mouse models. A-D) The top 20 enriched ontology clusters are shown for **A)** Dp16, **B)** Ts65Dn, **C)** Ts66Yah, and **D)** Ts1Cje. To generate these graphs, the significant pathways (defined by GO, terms hallmark gene sets, and other canonical pathways evaluated by Metascape) are hierarchically clustered based on similarities in gene members. Each cluster is named by its most significant pathway. Full pathway lists for each cluster are shown in Tab. S4.

Biology Open • Supplementary information

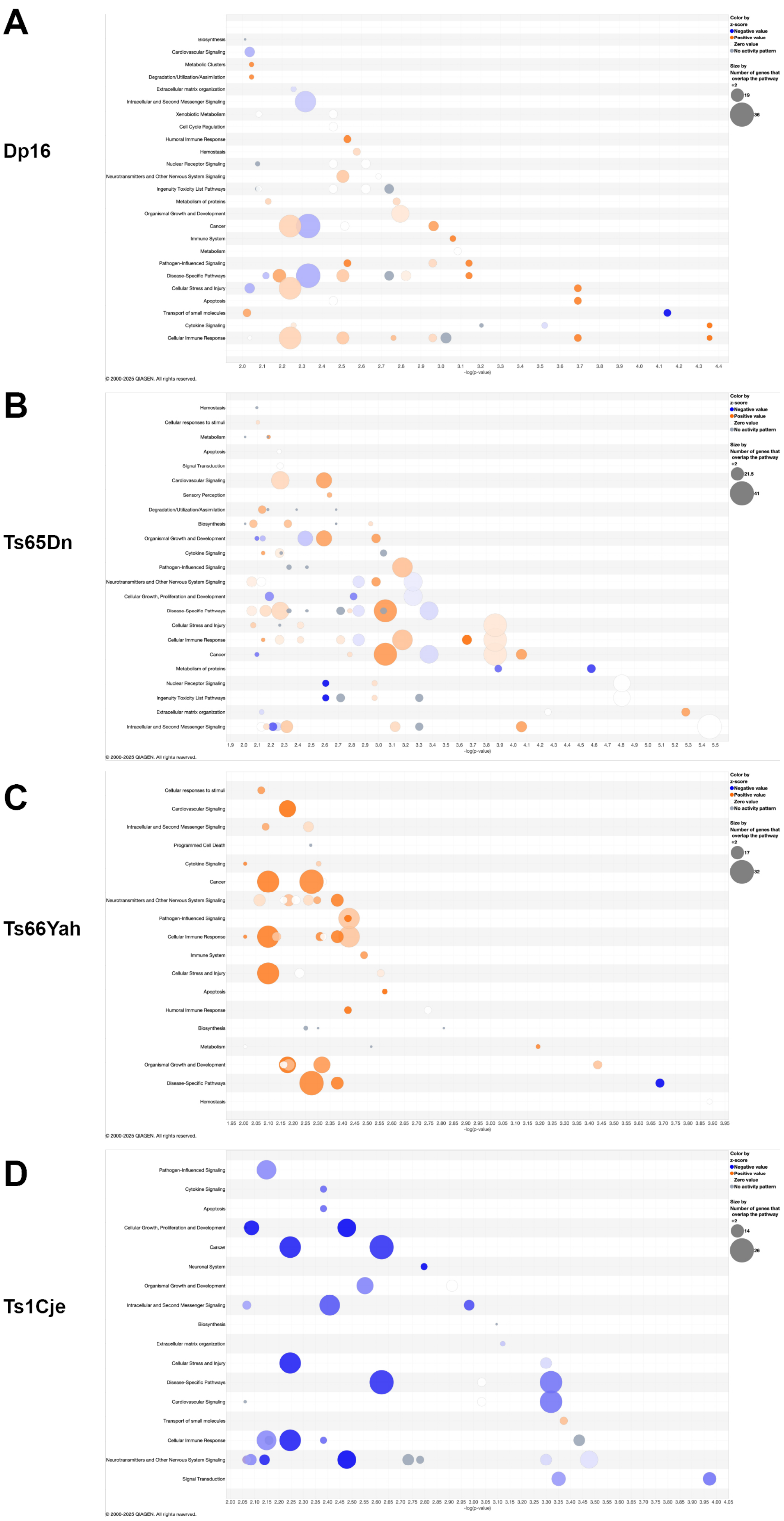

Fig. S3. Ingenuity pathway analysis functional clustering for placental MDGs in Dp16, Ts65Dn, Ts66Yah, and Ts1Cje mouse models. A-D) Ingenuity pathway analysis bubble charts are shown for the top ontology clusters in **A)** Dp16, **B)** Ts65Dn, **C)** Ts66Yah, and **D)** Ts1Cje. For each chart, the x-axis = the –Log10(*p*-value) for each pathway, and the y-axis indicates pathway categories. Bubble size indicates gene overlap, and color indicates Z-score value (orange = positive, blue = negative, white = zero, gray = no activity pattern).

Biology Open • Supplementary information

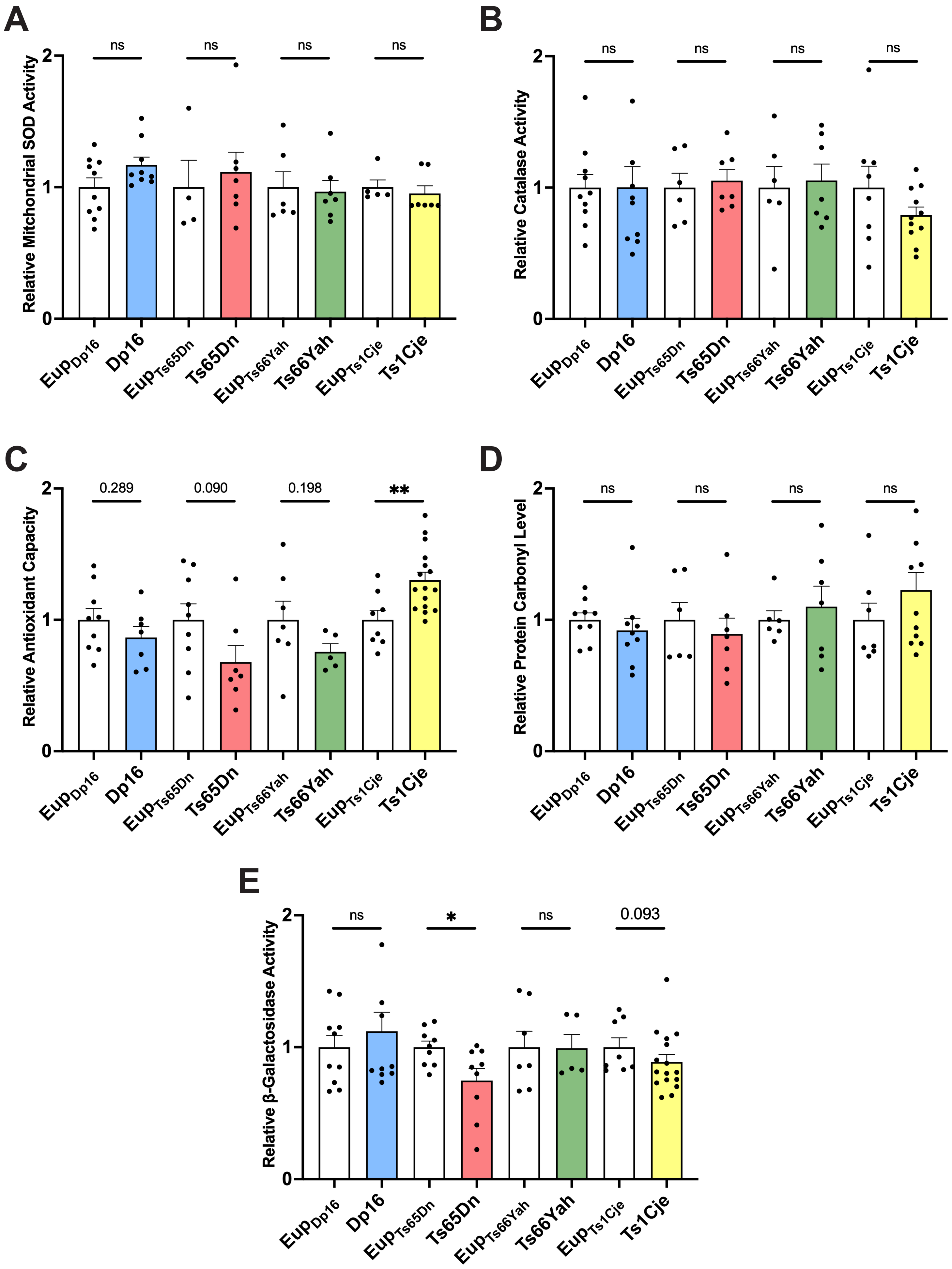

Biology Open • Supplementary information

Fig. S4. Oxidative stress and senescence assays in placentas of four mouse models of Down syndrome. A) Mitochondrial SOD activity. Sample numbers: Dp16 = 9 trisomic, 10 euploid; Ts65Dn = 7 trisomic, 4 euploid; Ts66Yah = 7 trisomic, 6 euploid; Ts1Cje = 7 trisomic, 5 euploid. **B)** Catalase activity. Sample numbers: Dp16 = 10 trisomic, 10 euploid; Ts65Dn = 7 trisomic, 6 euploid; Ts66Yah = 7 trisomic, 6 euploid; Ts1Cje = 11 trisomic, 8 euploid. **C)** Total antioxidant capacity (TAC). Sample numbers: Dp16 = 7 trisomic, 9 euploid; Ts65Dn = 7 trisomic, 9 euploid; Ts66Yah = 7 trisomic, 5 euploid; Ts1Cje = 16 trisomic, 8 euploid. **D)** Protein carbonyl level. Sample numbers: Dp16 = 9 trisomic, 9 euploid; Ts65Dn = 7 trisomic, 6 euploid; Ts66Yah = 7 trisomic, 6 euploid; Ts1Cje = 11 trisomic, 9 euploid. **E)** β-galactosidase activity. Sample numbers: Dp16 = 10 trisomic, 10 euploid; Ts65Dn = 9 trisomic, 9 euploid; Ts66Yah = 5 trisomic, 7 euploid; Ts1Cje = 16 trisomic, 8 euploid. For B, each sample was tested in duplicate, and for A and C-E, each sample was tested in triplicate. The average for each sample is plotted and was used for statistical analyses. Error bars = SEM. Unpaired, two-tailed t tests were performed for datasets with normal distribution, and two-tailed Mann-Whitney tests for datasets with non-normal distribution. ns, not significant, * p-value < 0.05, ** p-value < 0.01.

Biology Open • Supplementary information

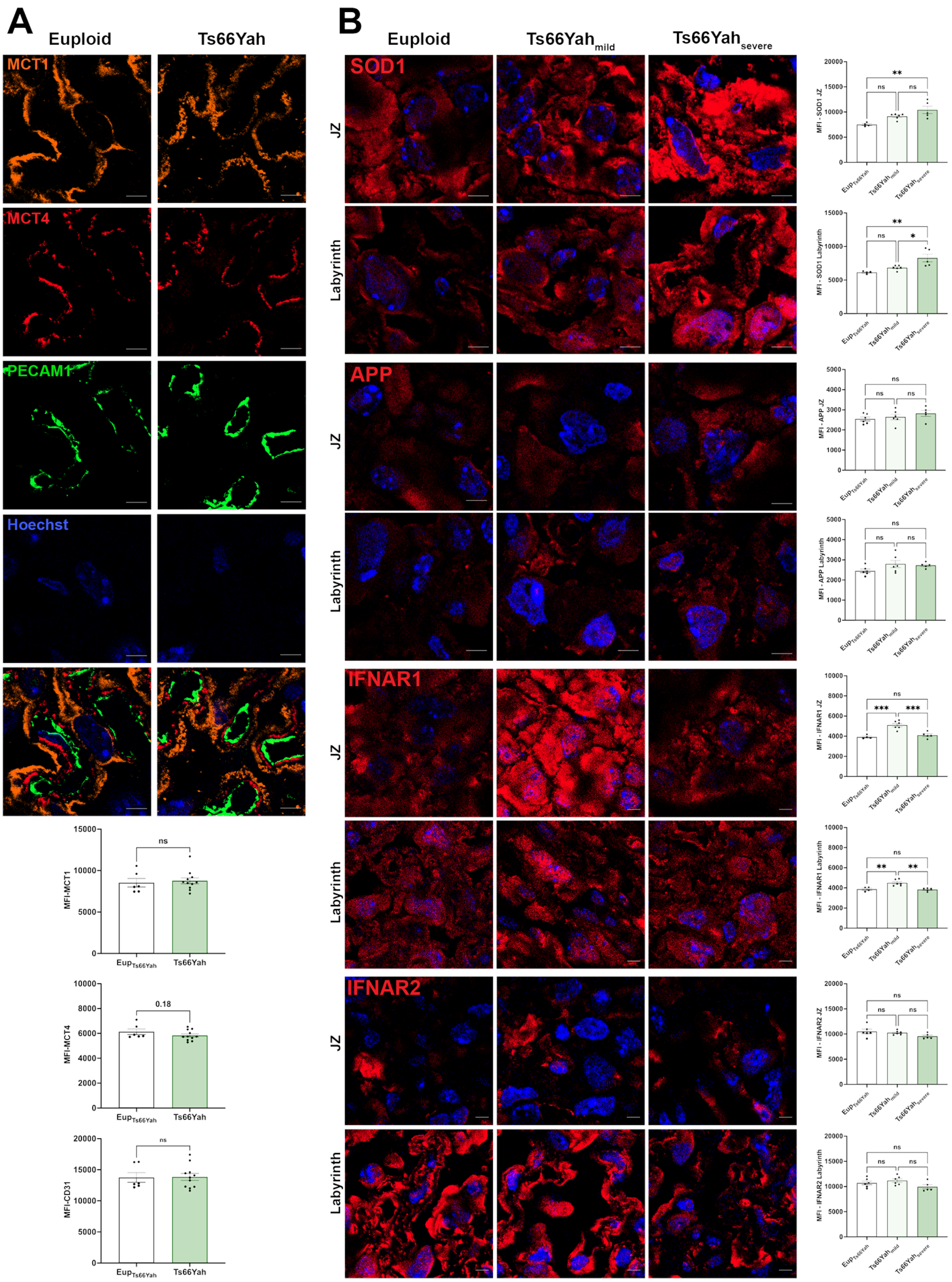

Biology Open • Supplementary information

Fig. S5. **Immunofluorescence staining of euploid and trisomic Ts66Yah placentas. A)** Immunofluorescence staining of MCT1, MCT4, and PECAM1/CD31 in the labyrinth of euploid and trisomic Ts66Yah placentas. Orange = MCT1 (syncytiotrophoblast-I), red = MCT4 (syncytiotrophoblast-II), green = PECAM1/CD31 (endothelial cells), and blue = Hoechst/nuclei. Error bars = SEM. A trend towards reduced expression of MCT4 was seen in trisomic placentas: Mean fluorescence intensity (MFI) = 6138 ± 565 in euploid vs 5843 ± 420 in Ts66Yah, *p*-value = 0.18 (Mann-Whitney test). Graphs (bottom) show the MFI of each target protein. **B)** Examination of SOD1, APP, IFNAR1, and IFNAR2 expression in the Junctional zone (JZ) and labyrinth of $Ts66Yah_{mild}$ and $Ts66Yah_{severe}$ placentas. Red = SOD1, APP, IFNAR1, or IFNAR2 and blue = Hoechst/nuclei. Graphs show the MFI of each target protein in each placental subregion. Error bars = SEM. One-way ANOVA analyses with Tukey's multiple comparison tests were performed, and adjusted p-values are reported. ns, not significant, * *p*-value < 0.05, ** *p*-value < 0.01, *** *p*-value < 0.001. Scale bar = 5 μm for all images.

Table S1. Differential gene expression analysis results for MDGs and all transcripts in the placenta of four mouse models of Down syndrome.

Available for download at
https://journals.biologists.com/bio/article-lookup/doi/10.1242/bio.062296#supplementary-data

Biology Open • Supplementary information

Table S2. Distribution of placental marginally dysregulated genes on autosomes in Dp16, Ts65Dn, Ts66Yah, and Ts1Cje.

	Dp16			Ts65Dn			Ts66Yah			Ts1Cje		
Chromosome (µarray probes)	Upregulated (%)	Downregulated (%)	Total (%)	Upregulated (%)	Downregulated (%)	Total (%)	Upregulated (%)	Downregulated (%)	Total (%)	Upregulated (%)	Downregulated (%)	Total (%)
Chr1 (1202)	2.25	2.16	4.41	2.00	1.91	3.91	2.75	1.16	3.91	1.00	1.75	2.75
Chr2 (1825)	1.48	2.30	3.78	2.25	1.97	4.22	2.68	0.60	3.29	1.10	1.15	2.25
Chr3 (1022)	1.57	2.05	3.62	1.47	2.64	4.11	2.25	2.05	4.31	1.17	1.76	2.94
Chr4 (1351)	2.22	2.00	4.22	1.85	2.00	3.85	2.37	1.18	3.55	1.41	1.63	3.03
Chr5 (1288)	1.86	1.86	3.73	1.79	2.02	3.80	2.17	0.93	3.11	1.09	1.09	2.17
Chr6 (1148)	1.57	2.26	3.83	2.18	1.66	3.83	1.66	0.87	2.53	1.31	1.83	3.14
Chr7 (2033)	1.87	1.97	3.84	2.12	1.77	3.89	2.36	1.72	4.08	0.79	1.43	2.21
Chr8 (1044)	1.15	2.11	3.26	1.63	2.20	3.83	1.15	1.44	2.59	1.25	1.25	2.49
Chr9 (1229)	2.44	1.63	4.07	2.12	2.12	4.23	1.55	0.81	2.36	1.30	1.79	3.09
Chr10 (1015)	2.17	1.67	3.84	1.48	2.17	3.65	2.27	1.28	3.55	0.59	1.18	1.77
Chr11 (1629)	0.98	2.89	3.87	2.15	2.15	4.30	2.21	1.60	3.81	1.29	1.66	2.95
Chr12 (675)	1.63	1.78	3.41	1.33	1.48	2.81	2.07	1.04	3.11	1.04	1.48	2.52
Chr13 (847)	2.24	2.24	4.49	3.07	2.01	5.08	3.31	2.01	5.31	1.42	1.77	3.19
Chr14 (923)	2.17	1.73	3.90	3.14	2.17	5.31	2.82	1.30	4.12	1.08	2.28	3.36
Chr15 (780)	1.67	1.67	3.33	1.41	1.67	3.08	2.31	1.41	3.72	0.77	1.41	2.18
Chr16 (674)	8.75	2.52	11.28	9.64	1.78	11.42	9.64	0.59	10.24	2.08	2.23	4.30
Chr17 (1069)	1.96	2.06	4.02	4.68	2.06	6.74	2.43	1.78	4.21	1.22	1.40	2.62
Chr18 (475)	1.05	2.53	3.58	2.11	2.32	4.42	2.95	0.21	3.16	0.84	1.26	2.11
Chr19 (710)	2.54	2.82	5.35	1.69	1.83	3.52	3.10	0.85	3.94	0.70	2.68	3.38

Table S3. (A) Mouse Genome Informatics query identified 279 placental MDGs in mouse models of Down syndrome that are associated with prenatal lethality, (B) Mouse Genome Informatics query identified 54 placental MDGs with previously identified placental phenotypes. (C) Placental Mammalian Phenotype Terms associated with the 54 MDGs with placental phenotypes. (D) A subset of the MDGs with placental mammalian phenotypes also have heart and brain phenotypes.

Available for download at
https://journals.biologists.com/bio/article-lookup/doi/10.1242/bio.062296#supplementary-data

Table S4. Metascape enriched pathway analyses for placental MDGs from Dp16, Ts65Dn, Ts66Yah, and Ts1Cje.

Available for download at
https://journals.biologists.com/bio/article-lookup/doi/10.1242/bio.062296#supplementary-data

Table S5. Enriched canonical pathways identified using Ingenuity Pathway Analysis for placental MDGs from Dp16, Ts65Dn, Ts66Yah, and Ts1Cje.

Available for download at
https://journals.biologists.com/bio/article-lookup/doi/10.1242/bio.062296#supplementary-data

Table S6. Predicted upstream regulators by Ingenuity Pathway Analysis of placental marginally dysregulated genes for four mouse models of Down syndrome.

			Dp16		Ts65Dn		Ts66Yah		Ts1Cje	
**Upstream Regulator**	**Molecule Type**	**Predicted Activation State**	**Bias-corrected z-score**	**p-value of overlap**	**Bias-corrected z-score**	**p-value of overlap**	**Bias-corrected z-score**	**p-value of overlap**	**Bias-corrected z-score**	**p-value of overlap**
TNF	cytokine	Activated	2.2	4.70E-11	2.0	1.64E-06	1.0	7.13E-08	2.5	7.80E-04
lipopolysaccharide	chemical drug	Activated	2.3	1.23E-11	2.7	1.55E-04	2.5	9.75E-12		
interferon alpha	group	Activated	4.4	9.27E-08			3.2	8.47E-09		
IFNAR	group	Activated	3.9	1.69E-06			2.4	1.33E-05		
IFN beta	group	Activated	3.8	4.04E-05			2.2	1.32E-07		
STING1	ion channel	Activated	3.7	1.95E-07			2.7	9.90E-05		
IRF9	transcription regulator	Activated	3.6	1.88E-08			2.2	1.60E-06		
SASH1	other	Activated	3.6	4.96E-06			2.0	7.00E-05		
SAMSN1	other	Activated	3.6	4.00E-05			2.1	3.68E-04		
IRF7	transcription regulator	Activated	3.3	2.11E-06			2.5	9.24E-05		
STAT1	transcription regulator	Activated	3.3	1.68E-05			3.5	1.58E-07		
IFNAR1	transmembrane receptor	Activated	3.2	1.76E-09			2.3	8.87E-08		
APP	other	Activated	3.0	9.30E-07			2.4	3.83E-05		
ZBTB10	transcription regulator	Activated	3.0	1.31E-05			3.0	1.13E-07		
TLR4	transmembrane receptor	Activated	2.9	6.61E-05			2.2	7.25E-06		
poly rI:rC-RNA	biologic drug	Activated	2.8	6.44E-09			3.1	3.28E-07		
DOCK8	other	Activated	2.8	2.46E-04			2.1	5.32E-04		
SENP3	peptidase	Activated	2.8	9.82E-07			2.1	3.16E-06		
CGAS	enzyme	Activated	2.7	8.06E-07			1.7	5.37E-05		
IFNB1	cytokine	Activated	2.5	1.39E-06			3.1	1.53E-05		
TICAM1	other	Activated	2.3	3.05E-05			2.6	3.12E-05		
IRGM1	enzyme	Inhibited	-4.2	6.18E-11			-2.7	5.80E-10		
TTC39A-AS1	other	Inhibited	-3.9	5.06E-12			-2.1	4.95E-05		
TRIM24	transcription regulator	Inhibited	-3.8	8.62E-11			-2.2	3.34E-05		
CITED2	transcription regulator	Inhibited	-3.6	8.09E-06			-3.3	6.55E-10		
TREX1	enzyme	Inhibited	-3.5	7.16E-07			-2.3	3.91E-09		
DUSP11	phosphatase	Inhibited	-3.4	8.67E-11			-1.7	9.65E-06		
PNPT1	enzyme	Inhibited	-3.0	2.72E-09			-2.2	7.30E-06		
RNASEH2B	other	Inhibited	-2.9	4.18E-08			-2.0	1.64E-06		
STAT6	transcription regulator	Inhibited	-2.6	2.31E-05			-2.2	1.26E-05		
SIRT1	transcription regulator	Inhibited	-2.4	9.63E-06			-2.6	5.72E-04		
PTGER4	g-protein coupled receptor	Inhibited	-2.2	1.65E-04			-2.7	1.75E-04		

Table S7a. Levels of Aβ-40 and Aβ-42 are unchanged in trisomic placentas relative to euploid controls.

	EupDp16	Dp16	p-value	EupTs65Dn	Ts65Dn	p-value	EupTs66Yah	Ts66Yah	p-value	EupTs1Cje	Ts1Cje	p-value
**AB40 (pg/mL)**	195.6 ± 26.63	169.9 ± 39.57	0.171	136.5 ± 36.81	150.5 ± 38.08	0.603	200 ± 70.54	184.3 ± 29.99	0.975	159.8 ± 25.65	168.4 ± 31.54	0.562
**AB42 (pg/mL)**	15.01 ± 1.99	13.79 ± 2.23	0.325	9.67 ± 1.41	10.30 ± 1.56	0.430	10.89 ± 1.37	12.37 ± 2.17	0.191	12.10 ± 1.59	12.16 ± 1.10	0.836
**AB-42/AB-40**	0.078 ± 0.014	0.086 ± 0.026	0.473	0.075 ± 0.022	0.071 ± 0.016	0.764	0.059 ± 0.018	0.068 ± 0.012	0.285	0.080 ± 0.022	0.073 ± 0.019	0.456

Table S7b. Levels of Aβ-40 and Aβ-42 in trisomic Ts66Yah mild and Ts66Yah severe placentas relative to euploid controls.

				p -value		
Amyloid Beta	$Eup_{Ts66Yah}$	$Ts66Yah_{mild}$	$Ts66Yah_{severe}$	**$Eup_{Ts66Yah}$ vs. $Ts66Yah_{mild}$**	**$Eup_{Ts66Yah}$ vs. $Ts66Yah_{severe}$**	**$Ts66Yah_{mild}$ vs. $Ts66Yah_{severe}$**
Aβ-40 (pg/mL)	200 ± 70.54	178.4 ± 31.87	194.3 ± 29.65	0.8843	0.903	0.903
Aβ-42 (pg/mL)	10.89 ± 1.37	11.1 ± 1.40	14.5 ± 1.32	0.9667	**0.0087**	**0.0152**
Aβ-42/Aβ-40	0.059 ± 0.018	0.064 ± 0.013	0.075 ± 0.005	0.6046	0.397	0.5411

Biology Open • Supplementary information

Table S8. Comparison of euploid and trisomic placental levels of cytokines, chemokines, STAT signaling proteins, and NF-kB signaling proteins.[1]

	EupDp16	Dp16	p-value	EupTs65Dn	Ts65Dn	p-value	EupTs66Yah	Ts66Yah	p-value	EupTs1Cje	Ts1Cje	p-value
**GM-CSF (pg/mL)**	4.254 ± 0.6063	3.74 ± 0.4754	0.080	3.006 ± 0.7053	3.04 ± 0	-	2.51 ± 0	2.841 ± 0.5752	-	3.236 ± 0.4655	3.683 ± 0.5318	0.224
**IFNG (pg/mL)**	336.8 ± 536.3	119.1 ± 162.8	0.467	70.05 ± 51.05	40.3 ± 31.15	0.103	193.2 ± 127.9	40.73 ± 55.18	0.034	44.34 ± 52.48	30.03 ± 42.58	0.788
**IL-1a (pg/mL)**	25.85 ± 14.33	23.22 ± 9.003	0.667	68 ± 117.9	24 ± 14.67	0.325	128.7 ± 242.6	20.78 ± 6.251	0.898	51.75 ± 36.25	29.74 ± 10.03	0.211
**IL-1b (pg/mL)**	0.7555 ± 0.1108	0.5092 ± 0.1575	0.005	0.4213 ± 0.1105	0.4267 ± 0.1122	0.917	0.566 ± 0.2062	0.4613 ± 0.1712	0.346	0.6618 ± 0.1623	0.7159 ± 0.1767	0.648
**IL-2 (pg/mL)**	1.533 ± 0.3917	1.288 ± 0.8227	0.460	1.65 ± 0.4383	1.824 ± 0.7391	0.740	2.816 ± 1.04	2.837 ± 1.058	>0.999	1.979 ± 0.9741	1.554 ± 0.4037	0.246
**IL-4 (pg/mL)**	1.236 ± 0.2807	1.176 ± 0.39	0.729	1.315 ± 0.2918	1.158 ± 0.4659	0.349	1.266 ± 0.2682	1.119 ± 0.3262	0.409	0.8693 ± 0.1972	1.34 ± 0.5525	0.027
**IL-5 (pg/mL)**	0.8513 ± 0.204	0.5461 ± 0.2587	0.026	0.9263 ± 0.2259	0.7224 ± 0.2298	0.023	1.302 ± 0.2525	1.18 ± 0.2894	0.446	1.106 ± 0.2651	1.076 ± 0.4427	0.869
**IL-6 (pg/mL)**	6.966 ± 1.494	8.188 ± 6.114	0.879	5.029 ± 1.662	3.4 ± 1.656	0.019	6.106 ± 2.081	5.633 ± 1.928	0.773	7.202 ± 2.067	6.031 ± 2.008	0.242
**IL-10 (pg/mL)**	9.071 ± 2.169	9.792 ± 2.511	0.549	13.67 ± 3.529	12.09 ± 3.19	0.288	17.09 ± 1.458	15.7 ± 3.889	0.179	9.378 ± 3.008	7.36 ± 2.294	0.179
**LIX (pg/mL)**	30.77 ± 0	30.44 ± 0	-	24.96 ± 4.304	28.02 ± 26.56	0.332	14.76 ± 7.511	13.95 ± 4.608	0.803	26.2 ± 8.15	22.58 ± 9.327	0.400
**IL-17a (pg/mL)**	0.1682 ± 0.0804	0.2586 ± 0.208	0.316	0.1484 ± 0.0784	0.2104 ± 0.1453	0.692	0.2214 ± 0.08816	0.2056 ± 0.1226	0.812	0.4131 ± 0.2608	0.3557 ± 0.3145	0.574
**KC (pg/mL)**	71.09 ± 21.04	66.41 ± 25.05	0.692	97.1 ± 22.14	97.82 ± 38.56	0.962	89.02 ± 36.1	69.48 ± 20.86	0.218	71.39 ± 16.02	64.54 ± 16.57	0.390
**MCP1 (pg/mL)**	19.13 ± 3.916	22.93 ± 18.48	0.235	19.34 ± 8.648	16.86 ± 6.082	0.506	32.05 ± 16.77	32.71 ± 16.6	0.797	20.84 ± 12.02	17.85 ± 4.748	0.480
**MIP2 (pg/mL)**	21.41 ± 3.801	19.84 ± 2.772	0.359	18.98 ± 1.415	19.11 ± 3.604	0.457	23.19 ± 4.143	21.8 ± 3.144	0.490	20.51 ± 4.081	23.52 ± 6.319	0.262
**TNFa (pg/mL)**	3.444 ± 0.4066	2.566 ± 0.7526	0.012	2.475 ± 1.2	1.958 ± 0.8681	0.286	4.026 ± 1.111	3.646 ± 0.9973	0.530	3.221 ± 0.7201	2.762 ± 0.6263	0.168
**STAT1 (MFI)**	4.844 ± 3.495	7.094 ± 2.835	0.179	3.857 ± 2.813	3.682 ± 1.881	0.875	2.938 ± 2.461	3.438 ± 2.382	0.741	17.63 ± 2.79	17.28 ± 3.681	0.827
**STAT3 (MFI)**	45.16 ± 11.06	42.84 ± 6.383	0.617	43.31 ± 7.929	38.83 ± 6.695	0.166	52.55 ± 13.36	53.33 ± 10.83	0.907	19.53 ± 7.413	22.4 ± 5.798	0.370
**STAT5 (MFI)**	7.813 ± 4.808	7.875 ± 3.12	0.627	17.94 ± 2.298	12.91 ± 6.59	0.052	19.6 ± 4.772	19.03 ± 5.688	0.853	7.094 ± 4.351	7 ± 2.984	0.948
**pNFkB (MFI)**	8.625 ± 2.553	8.25 ± 2.496	0.771	9.438 ± 3.453	8.45 ± 3.393	0.516	18.05 ± 2.96	16.28 ± 4.358	0.437	11.66 ± 3.27	10.35 ± 3.087	0.346
**TNFR1 (MFI)**	15.88 ± 9.459	18.84 ± 9.952	0.551	20.13 ± 8.035	13.05 ± 10.85	0.216	16.25 ± 9.985	30.33 ± 21.97	0.253	29.22 ± 6.856	23.13 ± 11.33	0.201
**cMyc (MFI)**	22.19 ± 8.051	17 ± 3.791	0.121	30.84 ± 12.05	18.47 ± 6.001	0.003	27.85 ± 5.222	23.11 ± 6.946	0.211	8.781 ± 3.924	9.15 ± 6.142	0.885
**pFADD (MFI)**	14.03 ± 3.421	13.41 ± 5.177	0.780	3.656 ± 1.647	3.55 ± 1.788	0.891	4.65 ± 1.318	5 ± 1.179	0.279	4.357 ± 1.632	3.679 ± 2.581	0.568
**IkkA/b (MFI)**	6.844 ± 1.118	6.5 ± 3.513	0.573	1.5 ± 1.541	2.708 ± 2.07	0.351	3.167 ± 3.643	2.125 ± 1.109	0.603	14.91 ± 3.226	9.175 ± 2.267	0.000
**IkB (MFI)**	159.2 ± 41.66	157 ± 20.35	0.893	95.97 ± 25.44	83.25 ± 20.2	0.203	150.9 ± 22.07	163.1 ± 42.97	0.568	74.75 ± 16.98	63.9 ± 26.61	0.333

[1]IL-13 could not be assessed due to insufficient detection of fluorescence; IKKα/β, TNFR1, STAT2, and STAT6 were excluded because cross-reactivity with mouse has not been validated.

Table S9. Probes for qPCR and antibodies for immunofluorescence and Western blots.

Gene Symbol	qPCR Assay ID, Thermo Fisher Scientific
Sod1	Mm01344233_g1
App	Mm01344172_m1
Ifnar1	Mm00439544_m1
Ifnar2	Mm00494916_m1
Clec1b	Mm00490925_m1
Prl4a1	Mm00478305_m1
Gapdh	Mm99999915_g1
Hprt	Mm00446968_m1

Antibody Target	Host	Vendor	Cat#	Concentration, Usage
SOD1	Rabbit	Abcam	ab51254	1:50, IF primary/ 1:50, Western
APP	Rabbit	Abcam	ab2072	1:50, IF primary/ 1:50, Western
IFNAR1	Rabbit	Thermo Fisher Scientific	MA5-32006	1:50, IF primary
IFNAR1	Rabbit	Thermo Fisher Scientific	BS-4116R	1:50, Western
IFNAR2	Rabbit	Thermo Fisher Scientific	PA5-76100	1:50, IF primary
MCT1	Chicken	Millipore	AB1286-I	1:300, IF primary
MCT4	Rabbit	Millipore	AB3314P	1:50, IF primary
PECAM1/CD31	Goat	R&D Systems	AF3628	1:15, IF primary
GAPDH	Mouse	Millipore	MAB374	1:200, Western
Anti rabbit-IgG Alexa-Fluor 647	Donkey	Thermo Fisher Scientific	A31573	1:200, IF secondary
Anti chicken-IgY Alexa-Fluor 555	Donkey	Thermo Fisher Scientific	A78949	1:200, IF secondary
Anti goat-IgY Alexa-Fluor 488	Donkey	Thermo Fisher Scientific	A11055	1:200, IF secondary
